# Associations of 10 dietary habits with breast cancer: a Mendelian randomization study

**DOI:** 10.3389/fnut.2023.1215220

**Published:** 2023-11-21

**Authors:** Xuyutian Wang, Lanlan Chen, Runxiang Cao, Ruolin Ma, Yutong Liu, Qian Zhao, Ye Du

**Affiliations:** ^1^Breast Surgery Department, General Surgery Center, First Hospital of Jilin University, Changchun, China; ^2^Hepatobiliary and Pancreatic Surgery Department, General Surgery Center, First Hospital of Jilin University, Changchun, China

**Keywords:** breast cancer, Mendelian randomization, dietary patterns, genome-wide association study (GWAS), nutrition

## Abstract

**Introduction:**

Epidemiological studies have revealed a link between dietary habits and the breast cancer risk. The causality of the association between food consumption and breast cancer requires further investigation.

**Methods:**

Using Mendelian randomization, we assessed the causal effects of 10 dietary habits on the risks of breast cancer and its subtypes (estrogen receptor [ER]  +  and ER- breast cancer). We obtained dietary pattern data in 2018 (number of single-nucleotide polymorphisms [SNPs]  =  9,851,867) and breast cancer data in 2017 (number of SNPs  =  10,680,257) from IEU OpenGWAS. Rigorous sensitivity analyses were conducted to ensure that the study results were credible and robust.

**Results:**

We identified that genetic predisposition to higher dried fruit intake was linked to a reduced risk of overall breast cancer (inverse variance-weighted [IVW] odds ratio [OR] = 0.55; 95% confidence interval [CI]: 0.43–0.70; *p* = 1.75 × 10^−6^), ER+ breast cancer (IVW OR = 0.62; 95% CI: 0.47–0.82; *p* = 8.96 × 10^−4^) and ER− breast cancer (IVW OR = 0.48; 95% CI: 0.34–0.68; *p* = 3.18 × 10^−5^), whereas genetic predisposition to more oily fish intake was linked to a lower risk of ER+ breast cancer (IVW OR = 0.73; 95% CI: 0.53–0.99; *p* = 0.04).

**Discussion:**

Our findings suggest that a genetic predisposition for dried fruit and oily fish consumption may be protective against breast cancer; however, further investigation is required.

## 1 Introduction

Among women, breast cancer is the most common type of cancer, accounting for 11.7% of all cancer cases, and the leading cause of cancer-related deaths (1). The existing epidemiological research broadly supports a link between nutrition and the cancer risk (2, 3). Elucidating the relationship between dietary factors and the breast cancer risk can help prevent breast cancer.

Several epidemiological studies have investigated the relationship between dietary patterns and breast cancer with conflicting findings. A series of prospective cohort studies have suggested an association between a reduced breast cancer risk and diets abundant in fruits, vegetables, cereals, fish, nuts, and olive oil (4–10). However, other studies have shown that these diets are not associated with the breast cancer risk (11–13). Considering the limitations of observational studies, the validity of these findings is subject to random and systematic errors, including the effects of cohort design bias, potential selection bias, small sample size, missed follow-ups, and the presence of reverse causality between outcomes and exposure (14). Randomized controlled trials are limited by ethical issues, cost, and long follow-ups (15). Whether or not these dietary patterns play a causal role in breast cancer remains unknown.

Mendelian randomization (MR) is an emerging method that infers the causality of exposure and outcome using single-nucleotide polymorphisms (SNPs) as instrumental variables (IV) for risk factors (16, 17). Random assignment of SNPs during meiosis and recombination mimic the random grouping of the randomized controlled trial experiments. Thus, in theory, this genetic tool is less susceptible to potential reverse causality and confounding bias (e.g., potential environmental factors). Using MR techniques, we investigated the potential link between 10 dietary patterns and breast cancer susceptibility (overall breast cancer and breast cancer stratified by estrogen receptor [ER] status).

## 2 Materials and methods

### 2.1 Study design

**Figure 1:**
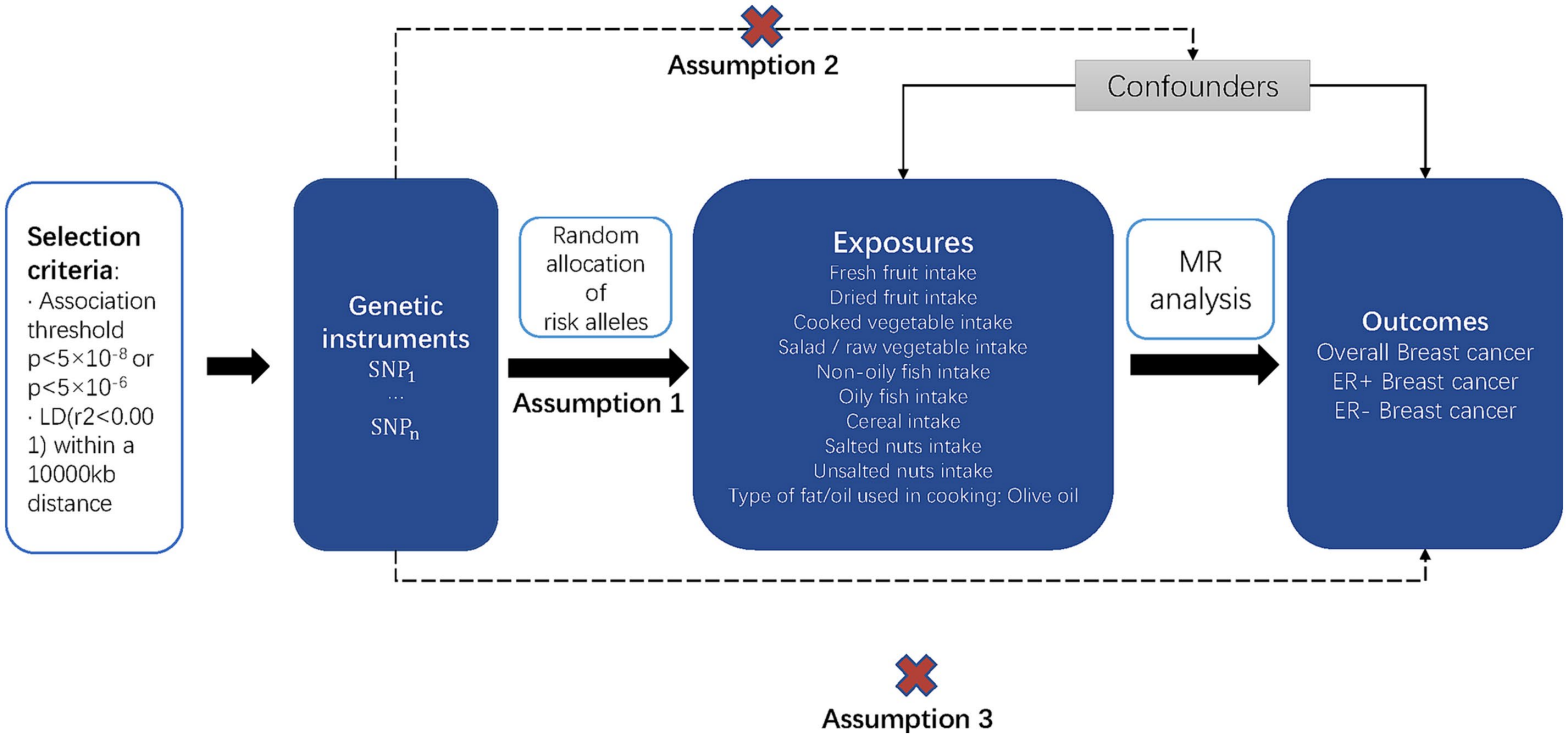
Overview of the study design and the three key assumptions of MR. Possible causal relationships between factors that might go against Mendelian randomization assumptions are shown by the dotted arrows.

We performed a two-sample MR analysis to investigate the causal impact of the risk factors on the outcomes. MR has the advantages of avoiding reverse causality and minimizing confounding effects in observational studies. Figure 1 shows a graphical flow of the experimental design.

As shown in Figure 1, the MR design is predicated on three key assumptions: assumption 1, IVs are strongly associated with exposure factors, indicated by the solid arrow in the figure; assumption 2, IVs are not associated with confounding factors, indicated by the dotted arrow in the figure; and assumption 3, the chosen IVs affect the outcome only via exposure, indicated by the dotted arrow in the figure (18).

### 2.2 GWAS summary data of 10 dietary habits and breast cancer

We collected exposure and outcome data from the IEU OpenGWAS database^1^, which contains 126 billion genetic associations from 14,582 complete GWAS datasets, representing human phenotypes and disease outcomes across different populations (19, 20). Table 1 shows the data source, sample size, number of SNPs, and strength of IVs for each exposure factor.

**Table 1:** Brief description of each exposure factor used in our study.

Year	Trait	Consortium	Author	Sample size	Number of SNPs	Number of IVs	*R* ^2^	*F*
2018	Fresh fruit intake	MRC-IEU	Ben Elsworth	446,462	9,851,867	55	0.001914	15.64080
2018	Dried fruit intake	MRC-IEU	Ben Elsworth	421,764	9,851,867	43	0.002523	24.74643
2018	Cooked vegetable intake	MRC-IEU	Ben Elsworth	448,651	9,851,867	17	0.000789	20.81829
2018	Salad / raw vegetable intake	MRC-IEU	Ben Elsworth	435,435	9,851,867	22	0.000861	17.04103
2018	Non-oily fish intake	MRC-IEU	Ben Elsworth	460,880	9,851,867	11	0.000657	27.54398
2018	Oily fish intake	MRC-IEU	Ben Elsworth	460,443	9,851,867	63	0.005204	38.03898
2018	Cereal intake	MRC-IEU	Ben Elsworth	441,640	9,851,867	43	0.003103	31.87728
2018	Salted nuts intake	MRC-IEU	Ben Elsworth	64,949	9,851,867	23	0.00074511	2.1040981
2018	Unsalted nuts intake	MRC-IEU	Ben Elsworth	64,949	9,851,867	16	0.001214852	4.931687334
2018	Type of fat/oil used in cooking: Olive oil	MRC-IEU	Ben Elsworth	64,949	9,851,867	8	0.000662498	5.378855561
2017	Breast cancer (combined Oncoarray; iCOGS; GWAS meta analysis)	BCAC	Michailidou K	228,951	10,680,257	-	-	-
2017	ER+ Breast cancer (combined Oncoarray; iCOGS; GWAS meta analysis)	BCAC	Michailidou K	175,475	10,680,257	-	-	-
2017	ER- Breast cancer (combined Oncoarray; iCOGS; GWAS meta analysis)	BCAC	Michailidou K	127,442	10,680,257	-	-	-

Exposure data were obtained from the United Kingdom Biobank GWAS database and aggregated using the IEU OpenGWAS (21). The consortium collected the 2018 statistics for fresh fruit (*n* = 446,462 participants), dried fruit (*n* = 421,764 participants), cooked vegetable (*n* = 448,651 participants), salad/raw vegetable (*n* = 435,435 participants), non-oily fish (*n* = 460,880 participants), oily fish (*n* = 460,443 participants), cereal (*n* = 441,640 participants), salted nut (*n* = 64,949 participants), unsalted nut (*n* = 64,949 participants), and olive oil (*n* = 64,949 participants) intake in the European population. Data on dietary patterns were obtained from the participants by answering a touchscreen questionnaire.

Outcome data, which are publicly available GWAS summary datasets of breast cancer, were obtained from the Breast Cancer Association Consortium. The consortium pooled statistics for the overall (*n* = 122,977 cases, 105,974 non-cases), ER+ (*n* = 69,501 cases), and ER- (*n* = 21,468 cases) breast cancer populations of European ancestry and adjusted the main covariates, including country and 10 principal components (22).

### 2.3 Statistical analysis

#### 2.3.1 IV selection

First, to extract SNPs genetically linked to the traits, we performed rigorous screening (*p* < 5 × 10^−8^; minor allele frequency [MAF] > 0.01) of SNPs associated with the dietary patterns. For the three dietary patterns of salted nut, unsalted nut, and olive oil intake, we did not select enough valid SNPs using the threshold of *p* < 5 × 10^−8^. To explore more associations between those three dietary patterns and breast cancer, we used the relatively relaxed threshold of *p* < 5 × 10^−6^ (23). Second, to remove linkage disequilibrium IVs, we excluded SNPs with *r*^2^ > 0.001, with the most significant SNPs within a clumping distance of 10,000 kb. Third, to remove incompatible and palindromic SNPs whose direction could not be determined, we harmonized the data. Data harmonization helps avoid redundant calculations of the same allele across datasets. Palindromic variants were removed by eliminating alleles with frequencies close to 50% (24). Fourth, to exclude weak IVs, we calculate the *F*-values using the formula $F=\frac{\left(N-k-1\right)R^{2}}{k\left(1-R^{2}\right)}$ (25). *R*^2^ in the formula was calculated using $R^{2}=2\times MAF\times \left(1-MAF\right)\times \mathrm{bet}a^{2}$ (26). When the F-statistic was <10, we determined that genetic variation to be a weak IV (27). We removed one SNP with an *F*-value <10 for fresh fruit intake (rs586346, *F* = 9.91). Table 1 lists the F- and R^2^ values for each exposure. Supplementary Table S1 shows the *F* and *R*^2^ values of all SNPs. Finally, to assess the statistical power of the results, we used http://cnsgenomic.com/shiny/mRnd/ (accessed on March 2, 2023) (28). Table 2 shows the calculated statistical power estimates.

**Table 2:** Estimation of power for Mendelian randomization analyses for breast cancer risk based on the total sample size and proportion of phenotypic variance of nutrients explained by instruments.

Outcome	Exposure	*N*	alpha	Proportion of cases	OR	*R* ^2^	power
Overall BC	Fresh fruit intake	228,951	0.05	0.537132	0.641836	0.001914	1
	Dried fruit intake	228,951	0.05	0.537132	0.540035	0.002523	1
	Cooked vegetable intake	228,951	0.05	0.537132	0.67135	0.000789	0.78
	Salad / raw vegetable intake	228,951	0.05	0.537132	0.880096	0.000861	0.15
	Non-oily fish intake	228,951	0.05	0.537132	0.430221	0.000657	1
	Oily fish intake	228,951	0.05	0.537132	0.744191	0.005204	1
	Cereal intake	228,951	0.05	0.537132	1.187451	0.003103	0.62
	Salted nuts intake	228,951	0.05	0.537132	1.246116975	0.00074511	0.30
	Unsalted nuts intake	228,951	0.05	0.537132	1.043704899	0.001214852	0.06
	Type of fat/oil used in cooking: olive oil	228,951	0.05	0.537132	0.716392316	0.000662498	0.55
ER+ Breast cancer	Fresh fruit intake	175,475	0.05	0.396074	0.650789	0.001914	0.99
	Dried fruit intake	175,475	0.05	0.396074	0.575736	0.002523	1
	Cooked vegetable intake	175,475	0.05	0.396074	0.701283	0.000789	0.62
	Salad / raw vegetable intake	175,475	0.05	0.396074	0.75149	0.000861	0.48
	Non-oily fish intake	175,475	0.05	0.396074	0.42784	0.000657	1
	Oily fish intake	175,475	0.05	0.396074	0.726119	0.005204	1
	Cereal intake	175,475	0.05	0.396074	1.285963	0.003103	0.92
	Salted nuts intake	175,475	0.05	0.396074	1.172298417	0.00074511	0.15
	Unsalted nuts intake	175,475	0.05	0.396074	1.157932392	0.001214852	0.19
	Type of fat/oil used in cooking: olive oil	175,475	0.05	0.396074	0.728121089	0.000662498	0.37
ER- Breast cancer	Fresh fruit intake	127,442	0.05	0.168453	0.550731	0.001914	0.97
	Dried fruit intake	127,442	0.05	0.168453	0.55935	0.002523	0.99
	Cooked vegetable intake	127,442	0.05	0.168453	0.504238	0.000789	0.79
	Salad / raw vegetable intake	127,442	0.05	0.168453	1.444774	0.000861	0.6
	Non-oily fish intake	127,442	0.05	0.168453	0.404211	0.000657	0.88
	Oily fish intake	127,442	0.05	0.168453	0.904957	0.005204	0.24
	Cereal intake	127,442	0.05	0.168453	0.88803	0.003103	0.21
	Salted nuts intake	127,442	0.05	0.168453	0.996131708	0.00074511	0.05
	Unsalted nuts intake	127,442	0.05	0.168453	1.179968583	0.001214852	0.13
	Type of fat/oil used in cooking: olive oil	127,442	0.05	0.168453	0.870739101	0.000662498	0.07

#### 2.3.2 MR analyses

This study was guided by the Strengthening the Reporting of Observational Research in Epidemiology using MR (29). We mainly used the inverse variance-weighted (IVW) model to evaluate the causal relationship between 10 dietary patterns and the breast cancer risk (including total breast cancer, ER+ breast cancer, and ER- breast cancer). The results of the MR-Egger and weighted median (WM) methods are also presented, which help interpret the results from multiple perspectives. To evaluate the sensitivity of the results, we performed Cochran’s Q, MR-Egger intercept, MR-PRESSO, and MR-Steiger filtering tests and plotted leave-one-out, scatter, and funnel plots. These plots are available in the Supplementary file. To assess the heterogeneity of the IVs, we performed Cochran’s Q test using IVW and MR-Egger’s methods. Heterogeneity was indicated when the *p*-value of the Cochran’s Q test was <0.05. When heterogeneity was strong, we used a random-effects model rather than a fixed-effects model (30). To test for the presence of horizontal pleiotropy, we calculated the difference between the MR-Egger intercept term and 0 (Pintercept). A significant difference (Pintercept <0.05) indicated the presence of horizontal pleiotropy. Supplementary Table S2 shows the results of Cochran’s Q and MR-Egger intercept tests. The MR-PRESSO method was used to exclude the influential outliers. To assess the direction of the effects of IV on the exposure and outcomes, we performed the MR-Steiger filtering test (31). Table 3 lists the outliers excluded by the MR-PRESSO and MR-Steiger filtering tests. To assess the robustness of the results, we constructed scatter plots and leave-one-out analyses.

**Table 3:** Outliers excluded by the MR-PRESSO and MR-Steiger filtering tests.

Trait	GWAS ID	Outcome	Outcome GWAS ID	Outliers	
				Steiger filtering	Mr-presso
Fresh fruit intake	ukb-b-3881	Overall BC	ieu-a-1126	NA	rs10828266, rs2143081, rs2867113, rs9919429
Dried fruit intake	ukb-b-16576	Overall BC	ieu-a-1126	NA	rs10740991, rs2328887
Cooked vegetable intake	ukb-b-8089	Overall BC	ieu-a-1126	rs1421085	rs1421085
Salad / raw vegetable intake	ukb-b-1996	Overall BC	ieu-a-1126	rs6482190	rs6482190, rs34186148
Non-oily fish intake	ukb-b-17627	Overall BC	ieu-a-1126	rs56094641	rs56094641, rs7148387
Oily fish intake	ukb-b-2209	Overall BC	ieu-a-1126	rs1421085	rs1421085, rs10828250, rs16891727, rs1876245, rs4510068
Cereal intake	ukb-b-15926	Overall BC	ieu-a-1126	rs9846396	rs9846396, rs1853931
Salted nuts intake	ukb-b-15960	Overall BC	ieu-a-1126	NA	NA
Unsalted nuts intake	ukb-b-12217	Overall BC	ieu-a-1126	NA	NA
Type of fat/oil used in cooking: olive oil	ukb-b-3875	Overall BC	ieu-a-1126	NA	NA
Fresh fruit intake	ukb-b-3881	ER+ Breast cancer	ieu-1-1127	rs10828266, rs9919429	rs10828266, rs9919429
Dried fruit intake	ukb-b-16576	ER+ Breast cancer	ieu-a-1127	rs7916868, rs10740991	rs7916868, rs10740991, rs2328887
Cooked vegetable intake	ukb-b-8089	ER+ Breast cancer	ieu-a-1127	rs1421085	rs1421085
Salad / raw vegetable intake	ukb-b-1996	ER+ Breast cancer	ieu-1-1127	rs6482190	rs6482190, rs34186148
Non-oily fish intake	ukb-b-17627	ER+ Breast cancer	ieu-a-1127	rs56094641	rs56094641
Oily fish intake	ukb-b-2209	ER+ Breast cancer	ieu-a-1127	rs1876245, rs1421085, rs10828250	rs1421085, rs10828250, rs16891727, rs1876245, rs4510068
Cereal intake	ukb-b-15926	ER+ Breast cancer	ieu-1-1127	rs9846396	rs9846396
Salted nuts intake	ukb-b-15960	ER+ Breast cancer	ieu-1-1127	NA	NA
Unsalted nuts intake	ukb-b-12217	ER+ Breast cancer	ieu-1-1127	NA	NA
Type of fat/oil used in cooking: olive oil	ukb-b-3875	ER+ Breast cancer	ieu-1-1127	NA	NA
Fresh fruit intake	ukb-b-3881	ER- Breast cancer	ieu-1-1128	rs12044599, rs1375566, rs149449, rs2867113, rs7818437	rs10828266, rs149449, rs2867113
Dried fruit intake	ukb-b-16576	ER- Breast cancer	ieu-a-1128	NA	rs10740991
Cooked vegetable intake	ukb-b-8089	ER- Breast cancer	ieu-1-1128	rs1421085	rs1421085
Salad / raw vegetable intake	ukb-b-1996	ER- Breast cancer	ieu-a-1128	NA	NA
Non-oily fish intake	ukb-b-17627	ER- Breast cancer	ieu-1-1128	rs56094641	rs56094641
Oily fish intake	ukb-b-2209	ER- Breast cancer	ieu-a-1128	rs1421085	rs10828250, rs1421085, rs45501495
Cereal intake	ukb-b-15926	ER- Breast cancer	ieu-1-1128	NA	NA
Salted nuts intake	ukb-b-15960	ER- Breast cancer	ieu-1-1128	NA	NA
Unsalted nuts intake	ukb-b-12217	ER- Breast cancer	ieu-a-1128	NA	NA
Type of fat/oil used in cooking: olive oil	ukb-b-3875	ER- Breast cancer	ieu-1-1128	NA	NA

GWAS ID is the id from open GWAS.

Statistical analyses were performed using R 4.2.1 software using the “Two-sample MR” and “MR-PRESSO” packages. We adopted a Bonferroni-corrected threshold of *p* = 0.00167 (0.05/30) as a sign of a significant effect and 0.00167 < *p* < 0.05, as a sign of a suggestive association.

## 3 Results

**Figure 2:**
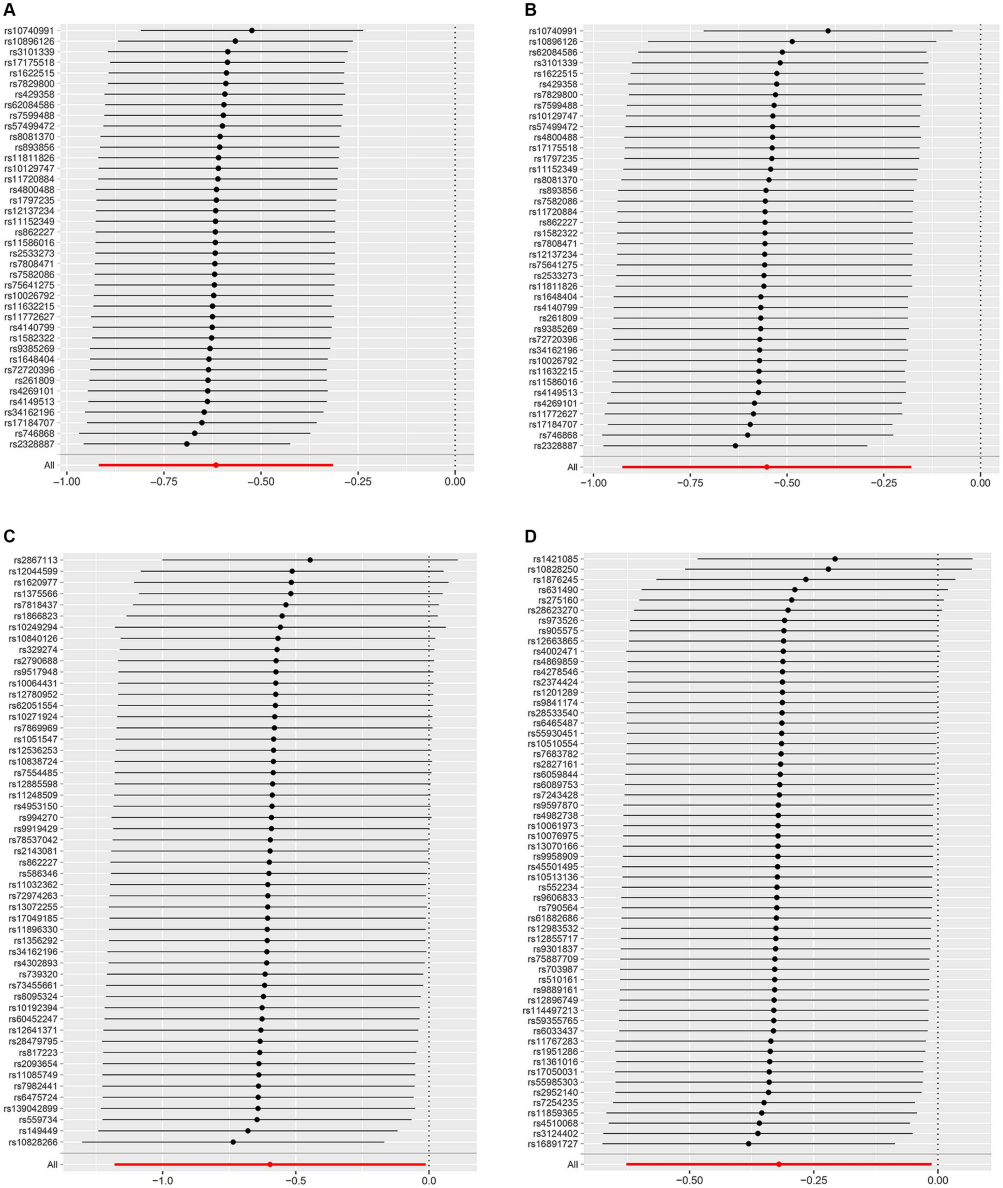
Leave-one-out plots of the MR results of **(A)** genetically predicted dried fruit intake with overall breast cancer; **(B)** genetically predicted dried fruit intake with ER+ breast cancer; **(C)** genetically predicted dried fruit intake with ER− breast cancer; **(D)** genetically predicted oily fish intake with ER+ breast cancer.

**Figure 3:**
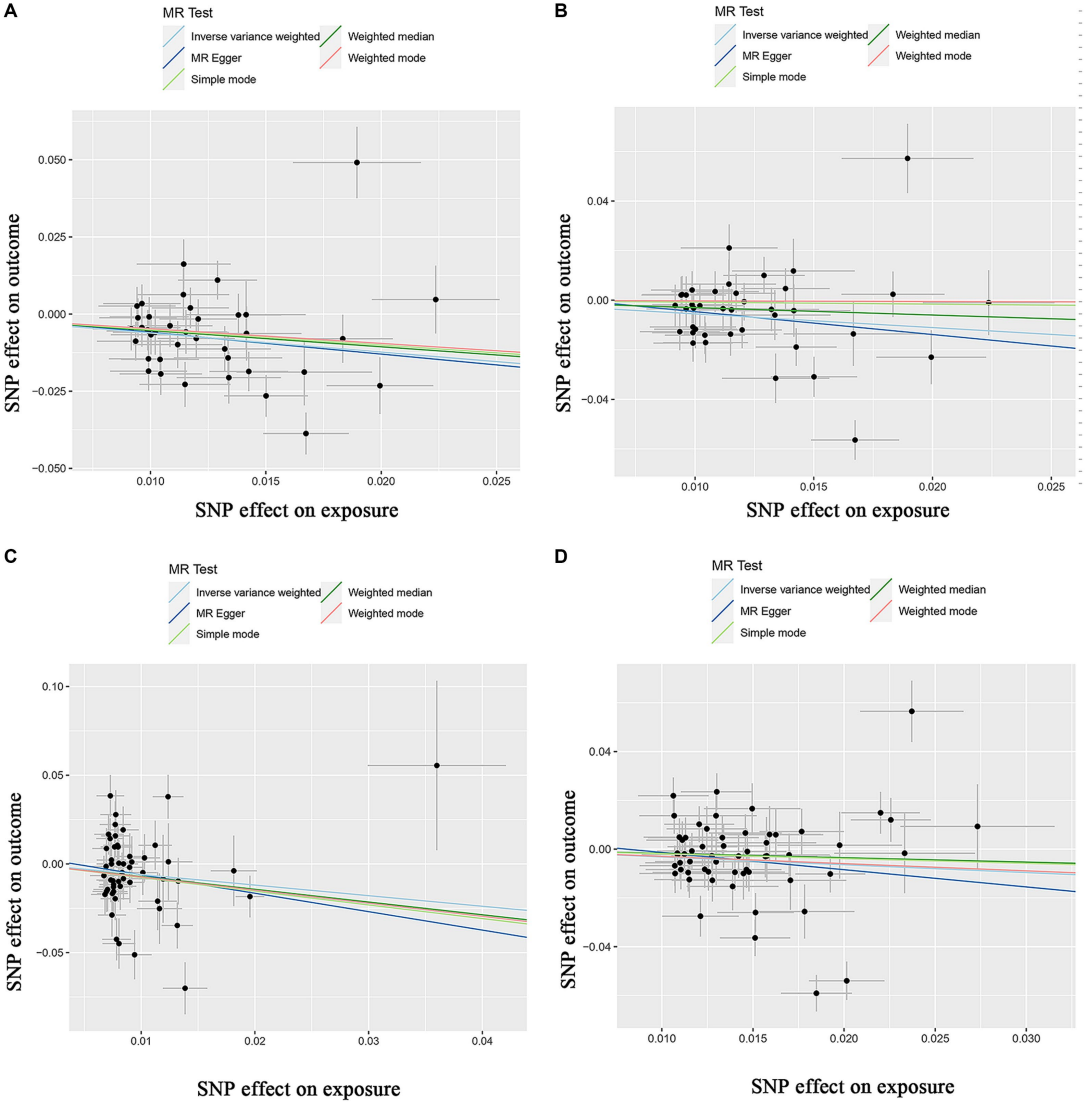
Scatter plots of the MR results of **(A)** genetically predicted dried fruit intake with overall breast cancer; **(B)** genetically predicted dried fruit intake with ER+ breast cancer; **(C)** genetically predicted dried fruit intake with ER− breast cancer; **(D)** genetically predicted oily fish intake with ER+ breast cancer.

**Figure 4:**
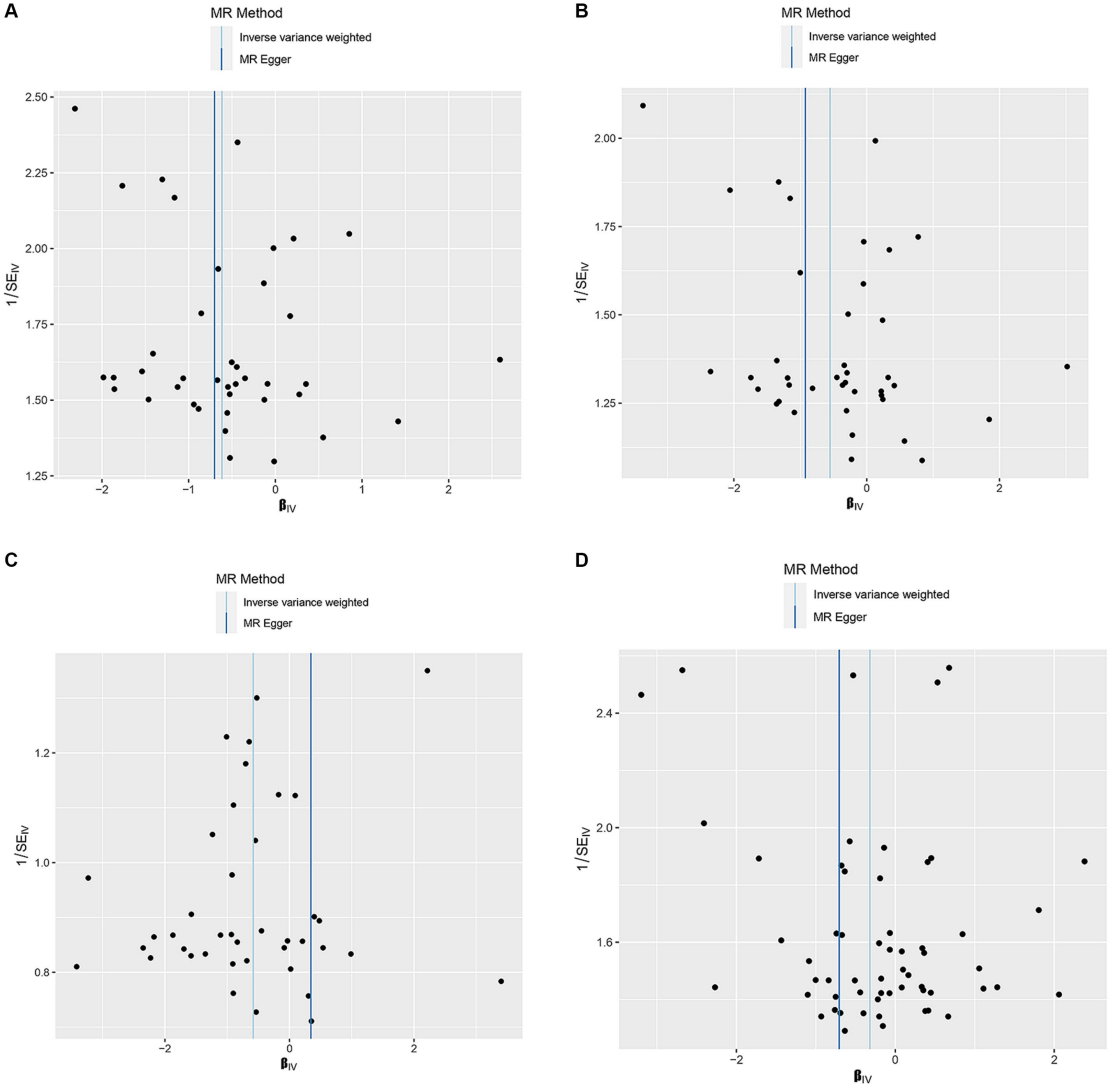
Funnel plots of the MR results of **(A)** genetically predicted dried fruit intake with overall breast cancer; **(B)** genetically predicted dried fruit intake with ER+ breast cancer; **(C)** genetically predicted dried fruit intake with ER− breast cancer; **(D)** genetically predicted oily fish intake with ER+ breast cancer.

Supplementary Table S1 shows the SNPs screened for strong associations with the 10 dietary factors. Table 3 shows the outliers excluded by the MR-PRESSO and MR-Steiger analyses. Heterogeneity was detected in all studies except for the dried fruit intake and ER- breast cancer study, salad/raw vegetable intake and ER- breast cancer study, and cereal intake and ER- breast cancer study; therefore, we applied a random-effects model in the IVW analysis. From the results of the pleiotropy test, we can assume the presence of no horizontal pleiotropy in terms of statistical significance because the *p*-values of the MR-Egger intercept were > 0.05. Figures 2–4 show leave-one-out, scatter, and funnel plots of MR for dietary patterns associated with the breast cancer risk based on the IVW analysis.

**Figure 5:**
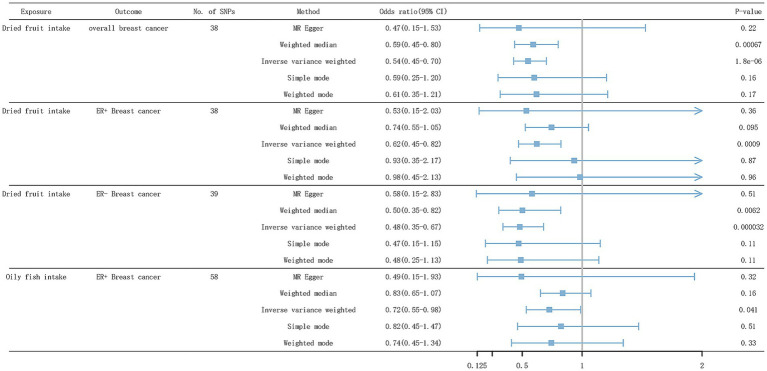
Forest plot of IVW results for dietary patterns associated with BC risk. SNP, single nucleotide polymorphism; ER+ breast cancer, Estrogen receptor-positive breast cancer; ER− breast cancer, Estrogen receptor-negative breast cancer.

As shown in Table 4 and Figure 5, after eliminating outliers based on MR-PRESSO and MR-Steiger filtering test results, of all dietary factors studied, only genetic tendency to intake dried fruit was found to be significantly associated with a reduced overall breast cancer risk (IVW odds ratio [OR] = 0.55; 95% confidence interval [CI]: 0.43–0.70; *p* = 1.75 × 10^−6^), ER+ breast cancer (IVW OR = 0.62; 95% CI: 0.47–0.82; *p* = 8.96 × 10^−4^) and ER- breast cancer (IVW OR = 0.48; 95% CI: 0.34–0.68; *p* = 3.18 ×10^−5^). In the WM model, dried fruit intake was associated with the overall breast cancer risk (WM OR = 0.59; 95% CI: 0.44–0.80; *p* = 6.68 × 10^−4^), and ER- breast cancer (WM OR = 0.50; 95% CI: 0.31–0.82; *p* = 6.22 × 10^−3^). However, in the WM model, dried fruit intake was not associated with ER+ breast cancer (WM OR = 0.74; 95% CI: 0.53–1.05; *p* = 0.09). Genetic tendency to intake oily fish, failing to pass the Bonferroni correction, was suggestive to be associated with a reduced risk of ER+ breast cancer (IVW OR = 0.73; 95% CI: 0.53–0.99; *p* = 0.04). In the WM model, oily fish intake was not associated with ER+ breast cancer (WM OR = 0.84; 95% CI: 0.65–1.07; *p* = 0.16).

**Table 4:** Association between genetic tendencies for 10 dietary habits and the breast cancer risk.

Exposure phenotypes		Number of SNPs	Overall breast cancer		Number of SNPs	ER+ Breast cancer		Number of SNPs	ER− Breast cancer	
			OR (95% CI)	*p*-value		OR (95% CI)	*p*-value		OR (95% CI)	*p*-value
Fruit	Fresh fruit intake	48	0.77 (0.58–1.01)	0.057242197	51	0.80 (0.59–1.09)	0.159731	47	0.65 (0.42–1.00)	0.050251382
	Dried fruit intake	38	0.55 (0.43–0.70)	1.75408 × 10^−6^	38	0.62 (0.47–0.82)	0.000896	39	0.48 (0.34–0.68)	3.18216 × 10^−5^
Vegetable	Cooked vegetable intake	17	0.67 (0.28–1.59)	0.363679601	17	0.70 (0.30–1.63)	0.410992	16	0.84 (0.43–1.67)	0.625276642
	Salad / raw vegetable intake	16	1.08 (0.70–1.69)	0.719768892	16	1.03 (0.64–1.67)	0.906074	18	1.44 (0.75–2.79)	0.27296844
Fish	Non-oily fish intake	8	0.71 (0.41–1.23)	0.223795105	9	0.79 (0.44–1.40)	0.414988	9	0.93 (0.46–1.91)	0.849043459
	Oily fish intake	53	0.85 (0.71–1.03)	0.091243395	58	0.73 (0.53–0.99)	0.04118	55	1.04 (0.77–1.41)	0.798353229
Cereal	Cereal intake	37	1.02 (0.82–1.29)	0.836510368	38	1.14 (0.88–1.48)	0.327498	39	0.89 (0.64–1.22)	0.468410676
Nuts	Salted nuts intake	22	1.25 (0.88–1.77)	0.219658057	22	1.17 (0.76–1.80)	0.467556675	22	1.00 (0.47–2.09)	0.991821678
	Unsalted nuts intake	15	1.04 (0.75–1.45)	0.799956254	15	1.16 (0.73–1.84)	0.537031221	15	1.18 (0.67–2.07)	0.562322459
Olive oil	Type of fat/oil used in cooking: olive oil	8	0.72 (0.48–1.08)	0.11128447	8	0.73 (0.45–1.18)	0.195888885	8	0.87 (0.45–1.68)	0.67906221

**Figure 6:**
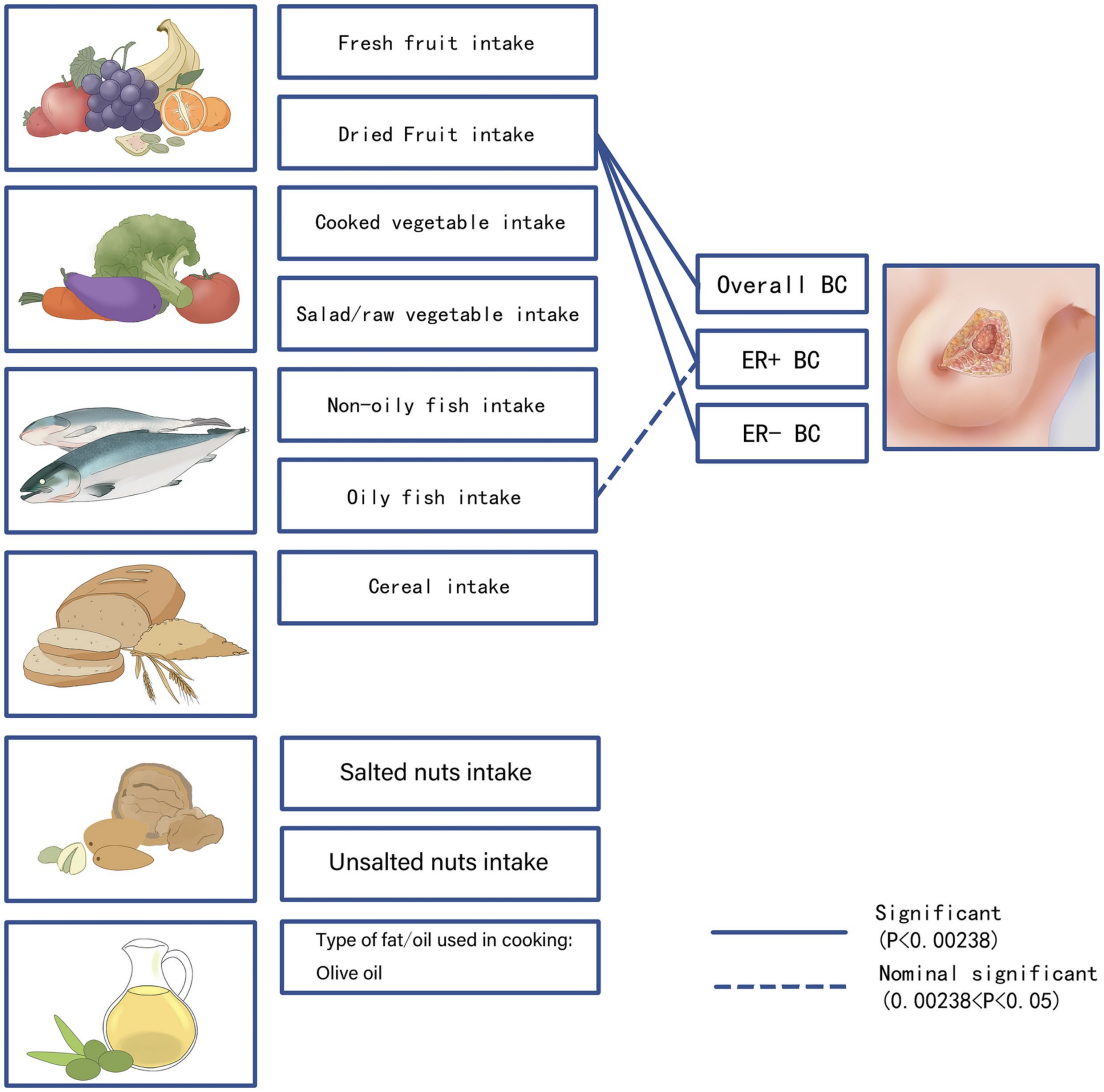
We visualized the association of 10 dietary patterns with breast cancer risk. *p*-values <0.00238 for the IVW method were considered as significant results, which we show as solid lines in the figure. 0.00238 <*p*-value<0.05 for the IVW method were considered as potentially significant results, which we show as dashed lines in the figure. BC, breast cancer; ER+ BC, Estrogen receptor-positive breast cancer; ER− BC, Estrogen receptor-negative breast cancer.

Fresh fruit (IVW OR = 0.77; 95% CI: 0.58–1.00; *p* = 0.05), cooked vegetable (IVW OR = 0.67; 95% CI: 0.28–1.59; *p* = 0.36), salad/raw vegetable (IVW OR = 1.08; 95% CI: 0.70–1.69; *p* = 0.72), non-oily fish (IVW OR = 0.71; 95% CI: 0.41–1.23; *p* = 0.22), oily fish (IVW OR = 0.85; 95% CI: 0.71–1.03; *p* = 0.09), cereal (IVW OR = 1.02; 95% CI: 0.82–1.29; *p* = 0.83), salted nut (IVW OR = 1.24; 95% CI: 0.87–1.77; *p* = 0.21), unsalted nut (IVW OR = 1.04; 95% CI: 0.74–1.45; *p* = 0.79), or olive oil (IVW OR = 0.71; 95% CI: 0.47–1.08; *p* = 0.11) was not associated with the overall breast cancer risk (Figure 6).

Fresh fruit (IVW OR = 0.80; 95% CI: 0.59–1.08; *p* = 0.15), cooked vegetable (IVW OR = 0.70; 95% CI: 0.30–1.63; *p* = 0.41), salad/raw vegetable (IVW OR = 1.02; 95% CI: 0.63–1.66; *p* = 0.90), non-oily fish (IVW OR = 0.78; 95% CI: 0.43–1.40; *p* = 0.41), cereal (IVW OR = 1.13; 95% CI: 0.87–1.47; *p* = 0.32), salted nut (IVW OR = 1.17; 95% CI: 0.76–1.80; *p* = 0.46), unsalted nut (IVW OR = 1.15; 95% CI: 0.72–1.84; *p* = 0.53), or olive oil (IVW OR = 0.72; 95% CI: 0.45–1.17; *p* = 0.19) intake was not associated with ER+ breast cancer.

Fresh fruit (IVW OR = 0.65; 95% CI: 0.42–1.00; *p* = 0.05), cooked vegetable (IVW OR = 0.84; 95% CI: 0.42–1.66; *p* = 0.62), salad/raw vegetable (IVW OR = 1.44; 95% CI: 0.74–2.78; *p* = 0.27), non-oily fish (IVW OR = 0.93; 95% CI: 0.45–1.90; *p* = 0.84), oily fish (IVW OR = 1.04; 95% CI: 0.76–1.41; *p* = 0.79), cereal (IVW OR = 0.88; 95% CI: 0.64–1.22; *p* = 0.46), salted nut (IVW OR = 0.99; 95% CI: 0.47–2.09; *p* = 0.99), unsalted nut (IVW OR = 1.17; 95% CI: 0.67–2.06; *p* = 0.56), or olive oil (IVW OR = 0.87; 95% CI: 0.45–1.67; *p* = 0.67) intake was not associated with ER- breast cancer.

The full MR results for the 10 dietary habits and overall, ER+, and ER- breast cancer risks, including results of the MR-Egger, WM, IVW, simple model, and WM methods, can be viewed in Supplementary Tables S3–S5, respectively.

## 4 Discussion

Observational studies lack correction for risk factors and have a range of biases, such as study design and population, which may lead to inconsistent conclusions. In a previous study, the causal relationship between dietary patterns and the breast cancer risk was not fully elucidated. Previous studies have not been uniformly conclusive regarding the association between fruit intake and the breast cancer risk in European populations. A subset of observational studies showed no significant association between fruit intake and the breast cancer risk (4, 5, 32). A European Prospective Investigation into Cancer and Nutrition study involving 285,526 women showed no significant association between fruit intake (including dried and canned fruits) and the breast cancer risk (33). However, a prospective nurses’ health study involving 182,145 women with >20 years of follow-up showed that a higher total fruit intake was associated with a reduced breast cancer risk (34). Clinical observational evidence is limited on the effect of dried fruit intake on the breast cancer risk. A prospective cohort study involving women in the United Kingdom found no association between dried fruit consumption and breast cancer incidence (35). The relationship between vegetable intake and the breast cancer risk has been controversial in previous observational studies. Previous epidemiological studies suggested that vegetable intake is not associated with the breast cancer risk (12, 36), whereas traditional studies have shown that vegetable intake is associated with the breast cancer risk (4, 5, 34, 37). The results of previous conventional studies on the relationship between grain intake and the breast cancer risk were conflicting. Some studies have found a negative association (38–42), while others have found no clear association (11, 43–45). Xiao et al. (6) previously conducted a meta-analysis of four cohort studies and seven case–control studies and observed a negative association between cereal intake and breast cancer only in the case–control study, while no negative association was observed in the cohort studies.

In this study, we used a large-scale GWAS database for a two-sample MR analysis and found that genetically predicted dried fruit intake plays a critical role in breast cancer susceptibility. In addition to dried fruit intake, we found evidence that genetic predisposition to greater intake of oily fish may reduce the risk of ER+ breast cancer. Our study had several strengths. First, we used a large-scale GWAS database, which allowed us to use a much larger sample size compared to traditional studies, thus minimizing bias. Second, our study population was from Europe, effectively limiting population heterogeneity bias. Third, using genetic variables associated with a single phenotype as IVs, we largely reduced the bias caused by common genetic effects between phenotypes. Fourth, we performed rigorous sensitivity analyses to assess the effects of outliers and pleiotropy. Fifth, our MR analysis assessed the causal effects of exposure and outcomes, thus reducing the reverse causality associated with the outcome. Sixth, the MR study allowed us to identify risk factors at the genetic level, and thus, early identification of high-risk groups, which had implications for disease screening. However, our study had certain limitations. First, all samples were from Europe, thus reducing the generalizability to other populations. Second, this study relied heavily on self-reporting and may have been subject to a reporting bias. Third, the MR results only suggest possible genetic correlations and causal associations at the genetic level, and more mechanism-based experiments are required to further confirm this biological plausibility (46). Although our findings clarify a causal relationship between some dietary patterns and the breast cancer risk, the causal relationships derived from the MR experiments cannot be fully equated to the expected impact of risk factors on outcomes in a clinical setting (47). Causal relationships in MR reflect a genetic-level predisposition to risk factors, which makes interventions for risk factors potentially clinically meaningful (46). Although guiding clinical interventions for risk factors based on MR results is not appropriate, causal inferences using MR designs may be useful for screening specific populations susceptible to disease and could provide some guidance for conducting randomized controlled trials. Fourth, for the three dietary patterns of salted nut, unsalted nut, and olive oil intake, we used relatively more relaxed threshold of *p* < 5 × 10^−6^, and the *F*-values of the SNPs were < 10, which may led to conclusions that would be relatively weakly IV biased, i.e., genetic variants may not be strongly correlated with exposure factors. This is due to the limited sample size of the exposures. In future studies, databases with larger sample sizes could help screen for more representative IVs. Fifth, the GWAS data on dietary patterns, particularly complex dietary patterns like the Mediterranean diet, are relatively limited. This represents a valuable direction for future GWAS databases and Mendelian randomization studies.

The potential mechanisms by which dried fruit and oily fish intake were associated with the breast cancer risk in this study should be discussed. Owing to thermal degradation and oxidation reactions, dried fruits contain higher amounts of nutrients and phytochemicals compared to fresh fruits. 5-hydroxymethylfurfural, a compound commonly found in dried fruits, exhibits beneficial biological properties including *in vitro* antioxidant activity (48) and anti-hypoxic effects (49). The processing of dried fruit may also reduce the cancer risk. Mycotoxins are exogenous toxins that may be generated during the processing of dried fruits. Low-dose mycotoxin consumption can activate physiological responses, thereby counteracting chronic inflammation (50). A recent review suggested that dried fruits can reduce the impact of carcinogens by inducing the detoxification of enzymes (51). Dried fruit was prepared by removing water from the fruit and had a nutrient profile similar to that of the equivalent fresh fruit but at higher concentrations. A comparative study on raisins and grapes showed that drying concentrated polyphenol content and thus increased antioxidant activity (52). Thus, microbiome metabolites of polyphenols and other phytonutrients may be beneficial to health (53). Polyphenols exert antioxidant effects that reduce the proliferation of cancer cells and protect the DNA from damage caused by carcinogens (54). In conclusion, dried fruit intake prevents breast cancer; however, the mechanism requires further investigation. Contrary to the relationship between fish intake and the breast cancer risk, previous studies have shown that fish intake was not associated with the breast cancer risk (13), unlike the findings of this experiment. A possible explanation is that although fish are rich in omega-3 polyunsaturated fatty acids, which retard breast cancer growth according to *in vitro* and animal studies (55). They may also be contaminated with dioxins, methylmercury, and PCBs (56, 57). The possible dangers of dioxins or other contaminants were outweighed by the health risks of not eating fish (58).

## 5 Conclusion

Our study revealed that distinct histological subtypes of breast cancer are affected by the genetic propensity to dry fruit and oily fish intake to varying degrees. These findings suggest that individuals who do not include dried fruits and oily fish in their diets may benefit from considering earlier or more frequent breast cancer screenings, such as breast ultrasound and mammography, to facilitate early breast cancer detection. Additionally, at the genetic level, our results indicate an association between dietary habits involving dried fruit and oily fish intake and a reduced breast cancer risk, thereby contributing to the value of dietary recommendations for breast cancer prevention.

## Data availability statement

The original contributions presented in the study are included in the article/Supplementary material, further inquiries can be directed to the corresponding author.

## Author contributions

XW and YD: conceptualization. XW: formal analysis and writing– original draft. YD: funding acquisition and supervision. XW and LC: software. LC, RC, RM, YL, and QZ: validation. XW, RC, RM, YL, and QZ: visualization. LC and YD: writing – review and editing. All authors contributed to the article and approved the submitted version.

## Footnotes

^1^https://gwas.mrcieu.ac=/, accessed on February 1, 2023.
