# Supplementary material for: Associations of 10 dietary habits with breast cancer: a Mendelian randomization study
Source: Front Nutr. 2023 Nov 21;10:1215220. doi: 10.3389/fnut.2023.1215220 (PMC10702979; doi:10.3389/fnut.2023.1215220)
Supplement: Supplementary file 1 [file Table_1.docx]

Supplementary Material

Associations of seven dietary habits related to Mediterranean diet with breast cancer: A Mendelian randomization study

**Xuyutian Wang^1^, Lanlan Chen^2^, Runxiang Cao^1^, Ruolin Ma^1^, Yutong Liu^1^, Qian Zhao^1^, Ye Du ^1^***

*** Correspondence:**Corresponding Author
jdyydy@jlu.edu.cn

# Supplementary Table

**Supplemental Table 1. Genetic associations with dietary habits**

| **Exposure phenotypes** | **SNP** | | **EA** | **OA** | **beta** | **eaf** | **se** | **p** | **r2** | **F** |
| --- | --- | --- | --- | --- | --- | --- | --- | --- | --- | --- |
| **fresh fruit intake** | 1 | rs2790688 | T | C | 0.0114466 | 0.154068 | 0.0016961 | 1.50E-11 | 3.4153E-05 | 15.248541 |
|  | 2 | rs559734 | C | G | 0.0077681 | 0.711817 | 0.0013608 | 1.10E-08 | 2.4757E-05 | 11.053127 |
|  | 3 | rs12044599 | G | A | 0.0094207 | 0.21008 | 0.0015033 | 3.70E-10 | 2.9456E-05 | 13.151106 |
|  | 4 | rs7554485 | C | T | -0.00801 | 0.611539 | 0.0012541 | 1.70E-10 | 3.0485E-05 | 13.61061 |
|  | 5 | rs1620977 | G | A | -0.013174 | 0.730517 | 0.0013776 | 1.10E-21 | 6.8335E-05 | 30.510718 |
|  | 6 | rs2867113 | A | G | -0.013861 | 0.130942 | 0.0019603 | 1.50E-12 | 4.3724E-05 | 19.521956 |
|  | 7 | rs17049185 | T | G | 0.0080404 | 0.267872 | 0.0013903 | 7.30E-09 | 2.5357E-05 | 11.321304 |
|  | 8 | rs11896330 | A | G | -0.008446 | 0.632839 | 0.0012744 | 3.40E-11 | 3.3147E-05 | 14.799428 |
|  | 9 | rs72974263 | T | C | 0.0073868 | 0.318282 | 0.0013124 | 1.80E-08 | 2.3679E-05 | 10.571998 |
|  | 10 | rs817223 | C | T | -0.007269 | 0.481314 | 0.0012234 | 2.80E-09 | 2.6383E-05 | 11.779204 |
|  | 11 | rs4953150 | T | C | -0.008439 | 0.344088 | 0.0012927 | 6.60E-11 | 3.2148E-05 | 14.353083 |
|  | 12 | rs10192394 | T | C | -0.007661 | 0.528785 | 0.0012287 | 4.50E-10 | 2.9248E-05 | 13.058397 |
|  | 13 | rs1375566 | A | G | -0.007834 | 0.627412 | 0.001266 | 6.10E-10 | 2.8692E-05 | 12.81014 |
|  | 14 | rs13072255 | C | A | 0.0089801 | 0.493966 | 0.0012236 | 2.10E-13 | 4.0315E-05 | 17.999942 |
|  | 15 | rs1356292 | T | C | 0.0091747 | 0.807546 | 0.0015532 | 3.50E-09 | 2.6164E-05 | 11.681613 |
|  | 16 | rs12641371 | T | C | 0.0079127 | 0.433148 | 0.0012335 | 1.40E-10 | 3.0746E-05 | 13.727214 |
|  | 17 | rs10064431 | C | T | -0.007574 | 0.522495 | 0.0012237 | 6.00E-10 | 2.8624E-05 | 12.779871 |
|  | 18 | rs149449 | A | G | 0.0072886 | 0.489167 | 0.0012206 | 2.40E-09 | 2.6549E-05 | 11.853387 |
|  | 19 | rs2143081 | A | G | 0.0083207 | 0.539822 | 0.0012289 | 1.30E-11 | 3.4398E-05 | 15.357721 |
|  | 20 | rs586346 | C | T | -0.006922 | 0.635353 | 0.0012658 | 4.50E-08 | 2.22E-05 | 9.9114106 |
|  | 21 | rs994270 | G | C | 0.0132585 | 0.235126 | 0.0014439 | 4.20E-20 | 6.3228E-05 | 28.230551 |
|  | 22 | rs329274 | G | A | 0.0068183 | 0.485636 | 0.0012281 | 2.80E-08 | 2.3225E-05 | 10.369464 |
|  | 23 | rs10271924 | T | C | -0.007047 | 0.492595 | 0.0012559 | 2.00E-08 | 2.4823E-05 | 11.082737 |
|  | 24 | rs10249294 | A | G | 0.0195637 | 0.372973 | 0.001263 | 4.10E-54 | 0.00017902 | 79.938488 |
|  | 25 | rs12536253 | C | G | -0.008159 | 0.24901 | 0.001416 | 8.30E-09 | 2.49E-05 | 11.117162 |
|  | 26 | rs7818437 | C | T | -0.008048 | 0.235903 | 0.0014525 | 3.00E-08 | 2.3351E-05 | 10.425497 |
|  | 27 | rs1866823 | A | G | 0.0074308 | 0.544469 | 0.0012406 | 2.10E-09 | 2.739E-05 | 12.228847 |
|  | 28 | rs6475724 | T | C | 0.0077216 | 0.727296 | 0.0013741 | 1.90E-08 | 2.3651E-05 | 10.559504 |
|  | 29 | rs7869969 | G | A | 0.0075674 | 0.330836 | 0.0012992 | 5.70E-09 | 2.5355E-05 | 11.320353 |
|  | 30 | rs4302893 | A | G | 0.0073886 | 0.334189 | 0.0012996 | 1.30E-08 | 2.4294E-05 | 10.846435 |
|  | 31 | rs2093654 | G | A | 0.0071292 | 0.388133 | 0.0012587 | 1.50E-08 | 2.414E-05 | 10.777949 |
|  | 32 | rs11248509 | T | A | 0.0073297 | 0.371237 | 0.001268 | 7.40E-09 | 2.5081E-05 | 11.197927 |
|  | 33 | rs9919429 | G | A | -0.006723 | 0.486008 | 0.0012229 | 3.80E-08 | 2.2583E-05 | 10.082699 |
|  | 34 | rs10828266 | G | A | 0.0123642 | 0.715634 | 0.001357 | 8.10E-20 | 6.222E-05 | 27.780512 |
|  | 35 | rs12780952 | A | G | 0.0074746 | 0.286382 | 0.0013536 | 3.40E-08 | 2.2836E-05 | 10.19545 |
|  | 36 | rs10840126 | G | A | -0.007718 | 0.376121 | 0.0012858 | 1.90E-09 | 2.7952E-05 | 12.47988 |
|  | 37 | rs10838724 | T | G | 0.0090109 | 0.367992 | 0.0012828 | 2.10E-12 | 3.7768E-05 | 16.862591 |
|  | 38 | rs11032362 | A | G | 0.0123929 | 0.090976 | 0.0021234 | 5.30E-09 | 2.5403E-05 | 11.34153 |
|  | 39 | rs60452247 | A | G | 0.0079697 | 0.363079 | 0.0012693 | 3.40E-10 | 2.9376E-05 | 13.115802 |
|  | 40 | rs7982441 | C | T | -0.008413 | 0.731883 | 0.0013761 | 9.80E-10 | 2.7775E-05 | 12.400781 |
|  | 41 | rs9517948 | T | C | 0.0069532 | 0.451352 | 0.0012329 | 1.70E-08 | 2.3944E-05 | 10.690434 |
|  | 42 | rs12885598 | A | G | 0.0075155 | 0.596671 | 0.0012474 | 1.70E-09 | 2.7186E-05 | 12.137614 |
|  | 43 | rs34162196 | T | C | -0.018142 | 0.1008 | 0.0020297 | 4.00E-19 | 5.9663E-05 | 26.638591 |
|  | 44 | rs28479795 | T | C | 0.0112324 | 0.221473 | 0.0014735 | 2.50E-14 | 4.3508E-05 | 19.425448 |
|  | 45 | rs62051554 | A | G | 0.0116118 | 0.108545 | 0.0019814 | 4.60E-09 | 2.6094E-05 | 11.650169 |
|  | 46 | rs862227 | G | A | -0.010145 | 0.457858 | 0.001224 | 1.10E-16 | 5.1097E-05 | 22.813916 |
|  | 47 | rs1051547 | C | T | -0.007575 | 0.561537 | 0.0012416 | 1.10E-09 | 2.8257E-05 | 12.616178 |
|  | 48 | rs139042899 | C | A | 0.035995 | 0.013469 | 0.0060811 | 3.20E-09 | 3.4432E-05 | 15.372976 |
|  | 49 | rs2048522 | T | A | 0.0095625 | 0.434968 | 0.0012477 | 1.80E-14 | 4.4947E-05 | 20.068057 |
|  | 50 | rs73455661 | G | A | 0.0102938 | 0.279338 | 0.0013622 | 4.10E-14 | 4.2662E-05 | 19.047772 |
|  | 51 | rs8095324 | G | A | -0.006946 | 0.404041 | 0.001249 | 2.70E-08 | 2.3236E-05 | 10.374096 |
|  | 52 | rs11085749 | A | G | -0.007734 | 0.387076 | 0.0012548 | 7.10E-10 | 2.8379E-05 | 12.670457 |
|  | 53 | rs2302593 | G | C | 0.008528 | 0.486625 | 0.001223 | 3.10E-12 | 3.6338E-05 | 16.223849 |
|  | 54 | rs739320 | C | T | -0.008997 | 0.60575 | 0.0012771 | 1.90E-12 | 3.8665E-05 | 17.263142 |
|  | 55 | rs78537042 | A | C | -0.011926 | 0.086801 | 0.0021843 | 4.80E-08 | 2.2548E-05 | 10.066878 |
| **Dried fruit intake** | 1 | rs261809 | G | A | -0.009629 | 0.540636 | 0.001679 | 9.80E-09 | 4.6049E-05 | 19.422443 |
|  | 2 | rs11586016 | C | G | 0.0098782 | 0.371004 | 0.0017303 | 1.10E-08 | 4.5542E-05 | 19.208682 |
|  | 3 | rs12137234 | T | C | 0.0102051 | 0.303772 | 0.0018374 | 2.80E-08 | 4.4052E-05 | 18.580198 |
|  | 4 | rs72720396 | G | A | 0.0114265 | 0.229157 | 0.0019854 | 8.70E-09 | 4.6127E-05 | 19.455526 |
|  | 5 | rs11811826 | A | T | 0.0132178 | 0.224231 | 0.0020058 | 4.40E-11 | 6.0782E-05 | 25.637181 |
|  | 6 | rs3101339 | C | A | 0.0142595 | 0.603285 | 0.0017055 | 6.20E-17 | 9.7328E-05 | 41.053431 |
|  | 7 | rs75641275 | C | A | -0.014161 | 0.143372 | 0.0023852 | 2.90E-09 | 4.926E-05 | 20.776945 |
|  | 8 | rs7582086 | T | G | -0.009628 | 0.468273 | 0.0016738 | 8.80E-09 | 4.6165E-05 | 19.471367 |
|  | 9 | rs7599488 | T | C | -0.010415 | 0.426408 | 0.0016873 | 6.70E-10 | 5.3065E-05 | 22.3821 |
|  | 10 | rs4149513 | A | G | 0.01173 | 0.493537 | 0.0016714 | 2.20E-12 | 6.8785E-05 | 29.012876 |
|  | 11 | rs17184707 | T | C | -0.011438 | 0.212811 | 0.0020401 | 2.10E-08 | 4.3834E-05 | 18.488316 |
|  | 12 | rs4269101 | G | T | -0.01381 | 0.718948 | 0.0018592 | 1.10E-13 | 7.7072E-05 | 32.508441 |
|  | 13 | rs11720884 | G | A | 0.0111797 | 0.250137 | 0.0019355 | 7.60E-09 | 4.6887E-05 | 19.775978 |
|  | 14 | rs57499472 | C | T | 0.0099123 | 0.404131 | 0.0017187 | 8.10E-09 | 4.7321E-05 | 19.959089 |
|  | 15 | rs10026792 | A | G | 0.0108465 | 0.290404 | 0.0018423 | 3.90E-09 | 4.8487E-05 | 20.450856 |
|  | 16 | rs1648404 | T | C | 0.009416 | 0.476112 | 0.0016736 | 1.80E-08 | 4.4229E-05 | 18.654883 |
|  | 17 | rs746868 | G | C | -0.012906 | 0.614662 | 0.0017145 | 5.20E-14 | 7.8897E-05 | 33.278181 |
|  | 18 | rs9385269 | T | C | 0.0120673 | 0.524565 | 0.0016818 | 7.20E-13 | 7.2634E-05 | 30.636537 |
|  | 19 | rs2328887 | C | T | 0.0189482 | 0.899467 | 0.0027761 | 8.80E-12 | 6.4932E-05 | 27.387692 |
|  | 20 | rs2533273 | A | C | -0.009877 | 0.48453 | 0.0016771 | 3.90E-09 | 4.8727E-05 | 20.552125 |
|  | 21 | rs7808471 | C | T | -0.011536 | 0.322136 | 0.001786 | 1.10E-10 | 5.8122E-05 | 24.514897 |
|  | 22 | rs11772627 | C | G | 0.0183338 | 0.18202 | 0.002171 | 3.00E-17 | 0.00010009 | 42.218975 |
|  | 23 | rs7829800 | G | A | -0.010446 | 0.671041 | 0.0017871 | 5.10E-09 | 4.8178E-05 | 20.32049 |
|  | 24 | rs10740991 | C | G | 0.016739 | 0.717606 | 0.0018572 | 2.00E-19 | 0.00011356 | 47.901301 |
|  | 25 | rs7916868 | T | A | 0.0096074 | 0.503499 | 0.0016717 | 9.10E-09 | 4.6149E-05 | 19.464752 |
|  | 26 | rs893856 | A | G | -0.013361 | 0.148988 | 0.0023492 | 1.30E-08 | 4.5268E-05 | 19.093344 |
|  | 27 | rs10896126 | G | A | -0.015009 | 0.303582 | 0.0018192 | 1.60E-16 | 9.5253E-05 | 40.178002 |
|  | 28 | rs11037497 | C | G | 0.0104396 | 0.446175 | 0.0016844 | 5.70E-10 | 5.3861E-05 | 22.717804 |
|  | 29 | rs1622515 | G | A | 0.0099172 | 0.484704 | 0.0016708 | 2.90E-09 | 4.9129E-05 | 20.721893 |
|  | 30 | rs3764002 | T | C | 0.0131215 | 0.261416 | 0.0019008 | 5.10E-12 | 6.6486E-05 | 28.043057 |
|  | 31 | rs4140799 | A | G | 0.0094566 | 0.531856 | 0.0016785 | 1.80E-08 | 4.4532E-05 | 18.782801 |
|  | 32 | rs34162196 | T | C | -0.022363 | 0.101001 | 0.0027717 | 7.10E-16 | 9.0817E-05 | 38.306646 |
|  | 33 | rs10129747 | G | A | 0.009359 | 0.530254 | 0.0016813 | 2.60E-08 | 4.3635E-05 | 18.404507 |
|  | 34 | rs1797235 | C | G | -0.010021 | 0.374623 | 0.0017424 | 8.90E-09 | 4.7049E-05 | 19.844569 |
|  | 35 | rs11632215 | C | A | -0.014143 | 0.120179 | 0.0025838 | 4.40E-08 | 4.2302E-05 | 17.842117 |
|  | 36 | rs862227 | G | A | -0.009165 | 0.458327 | 0.0016724 | 4.30E-08 | 4.1703E-05 | 17.58957 |
|  | 37 | rs1582322 | G | A | 0.0099435 | 0.604805 | 0.0017157 | 6.80E-09 | 4.7264E-05 | 19.935166 |
|  | 38 | rs62084586 | C | T | 0.0133946 | 0.165729 | 0.0022617 | 3.20E-09 | 4.9613E-05 | 20.9259 |
|  | 39 | rs8081370 | T | C | -0.016667 | 0.910232 | 0.002938 | 1.40E-08 | 4.5394E-05 | 19.146299 |
|  | 40 | rs4800488 | A | C | 0.0119836 | 0.489858 | 0.001672 | 7.70E-13 | 7.1774E-05 | 30.273631 |
|  | 41 | rs17175518 | A | C | 0.0114962 | 0.232775 | 0.0019751 | 5.90E-09 | 4.7206E-05 | 19.91066 |
|  | 42 | rs11152349 | A | G | 0.0099122 | 0.302856 | 0.0018176 | 4.90E-08 | 4.1488E-05 | 17.498865 |
|  | 43 | rs429358 | C | T | 0.0199445 | 0.154208 | 0.0023136 | 6.70E-18 | 0.00010376 | 43.768261 |
| **Cooked vegetable intake** | 1 | rs2252508 | G | A | 0.0091021 | 0.480256 | 0.0015622 | 5.70E-09 | 4.136E-05 | 18.556675 |
|  | 2 | rs2102738 | C | A | -0.012158 | 0.172403 | 0.002083 | 5.30E-09 | 4.218E-05 | 18.924688 |
|  | 3 | rs4851029 | G | T | 0.0101744 | 0.526969 | 0.0015642 | 7.80E-11 | 5.1609E-05 | 23.155353 |
|  | 4 | rs12629972 | C | T | 0.0117995 | 0.588311 | 0.0015908 | 1.20E-13 | 6.7442E-05 | 30.260035 |
|  | 5 | rs28450747 | A | G | -0.01016 | 0.23257 | 0.0018547 | 4.30E-08 | 3.6851E-05 | 16.533575 |
|  | 6 | rs1816263 | C | T | 0.0095812 | 0.280188 | 0.0017406 | 3.70E-08 | 3.7029E-05 | 16.613572 |
|  | 7 | rs2844672 | A | G | -0.009642 | 0.624125 | 0.0016092 | 2.10E-09 | 4.3621E-05 | 19.571244 |
|  | 8 | rs12550717 | A | G | 0.0091874 | 0.372413 | 0.001619 | 1.40E-08 | 3.9456E-05 | 17.702517 |
|  | 9 | rs10156602 | G | A | 0.0110063 | 0.361369 | 0.0016369 | 1.80E-11 | 5.5913E-05 | 25.086759 |
|  | 10 | rs11138705 | C | G | 0.0103715 | 0.757204 | 0.00183 | 1.40E-08 | 3.9552E-05 | 17.745631 |
|  | 11 | rs2052063 | T | C | -0.009458 | 0.515902 | 0.0015679 | 1.60E-09 | 4.4677E-05 | 20.045362 |
|  | 12 | rs349062 | C | G | -0.008904 | 0.39275 | 0.0015977 | 2.50E-08 | 3.7817E-05 | 16.96731 |
|  | 13 | rs28711392 | C | T | -0.010746 | 0.367132 | 0.0016322 | 4.60E-11 | 5.3662E-05 | 24.07671 |
|  | 14 | rs10161952 | C | A | -0.00958 | 0.31274 | 0.0016859 | 1.30E-08 | 3.945E-05 | 17.699916 |
|  | 15 | rs1421085 | C | T | 0.0103316 | 0.40346 | 0.0015905 | 8.30E-11 | 5.1381E-05 | 23.05336 |
|  | 16 | rs838133 | G | A | 0.0116877 | 0.549575 | 0.0016147 | 4.50E-13 | 6.763E-05 | 30.344057 |
|  | 17 | rs34155012 | T | C | 0.0105584 | 0.227386 | 0.0019221 | 3.90E-08 | 3.917E-05 | 17.574227 |
| **Salad / raw vegetable intake** | 1 | rs9427220 | T | A | -0.008008 | 0.554661 | 0.0014421 | 2.80E-08 | 3.1681E-05 | 13.795348 |
|  | 2 | rs4083969 | G | C | 0.017133 | 0.057217 | 0.0031152 | 3.80E-08 | 3.1669E-05 | 13.790142 |
|  | 3 | rs7619139 | A | T | 0.0124765 | 0.589193 | 0.0014509 | 8.00E-18 | 7.5355E-05 | 32.814442 |
|  | 4 | rs13102393 | G | C | 0.0079793 | 0.499077 | 0.0014303 | 2.40E-08 | 3.1835E-05 | 13.86234 |
|  | 5 | rs17460017 | T | A | 0.0111744 | 0.190124 | 0.0018134 | 7.20E-10 | 3.8453E-05 | 16.74449 |
|  | 6 | rs2194027 | A | T | -0.008608 | 0.484664 | 0.0014357 | 2.00E-09 | 3.7014E-05 | 16.117517 |
|  | 7 | rs3129962 | A | G | -0.013303 | 0.129168 | 0.0021226 | 3.70E-10 | 3.9814E-05 | 17.33711 |
|  | 8 | rs12203592 | T | C | -0.010286 | 0.219338 | 0.0016941 | 1.30E-09 | 3.6234E-05 | 15.778071 |
|  | 9 | rs3095337 | C | G | -0.012622 | 0.203909 | 0.0017664 | 9.00E-13 | 5.1722E-05 | 22.522443 |
|  | 10 | rs75248709 | T | C | -0.019734 | 0.045947 | 0.0035277 | 2.20E-08 | 3.4142E-05 | 14.866943 |
|  | 11 | rs57221424 | G | C | 0.008941 | 0.321733 | 0.0015333 | 5.50E-09 | 3.489E-05 | 15.192734 |
|  | 12 | rs62461186 | C | A | -0.011344 | 0.179827 | 0.0018573 | 1.00E-09 | 3.7958E-05 | 16.528952 |
|  | 13 | rs790561 | G | A | 0.012474 | 0.704134 | 0.001562 | 1.40E-15 | 6.4832E-05 | 28.231981 |
|  | 14 | rs7821179 | C | G | -0.010818 | 0.846577 | 0.0019764 | 4.40E-08 | 3.0403E-05 | 13.238767 |
|  | 15 | rs10819082 | A | G | -0.009165 | 0.66734 | 0.0015134 | 1.40E-09 | 3.7292E-05 | 16.238897 |
|  | 16 | rs6482190 | G | A | 0.0112719 | 0.719408 | 0.0015903 | 1.40E-12 | 5.1295E-05 | 22.336669 |
|  | 17 | rs1890012 | G | T | -0.010426 | 0.19473 | 0.0018079 | 8.10E-09 | 3.409E-05 | 14.844286 |
|  | 18 | rs12908495 | A | C | -0.00935 | 0.242533 | 0.0016657 | 2.00E-08 | 3.2121E-05 | 13.986951 |
|  | 19 | rs1052352 | T | C | 0.0081644 | 0.52355 | 0.0014256 | 1.00E-08 | 3.3255E-05 | 14.480851 |
|  | 20 | rs34186148 | C | G | -0.008051 | 0.370054 | 0.0014749 | 4.80E-08 | 3.0223E-05 | 13.160666 |
|  | 21 | rs4291983 | A | C | -0.0084 | 0.517576 | 0.0014248 | 3.70E-09 | 3.5235E-05 | 15.343087 |
|  | 22 | rs8130508 | A | G | 0.0087404 | 0.289686 | 0.001578 | 3.00E-08 | 3.1439E-05 | 13.690064 |
| **Non-oily fish intake** | 1 | rs16822430 | C | T | 0.0116344 | 0.23321 | 0.00192 | 1.40E-09 | 4.8411E-05 | 22.312518 |
|  | 2 | rs1260326 | C | T | -0.009554 | 0.604249 | 0.0016553 | 7.90E-09 | 4.3651E-05 | 20.118776 |
|  | 3 | rs11680516 | C | T | 0.0122842 | 0.202388 | 0.0020283 | 1.40E-09 | 4.8719E-05 | 22.454704 |
|  | 4 | rs3799077 | G | T | -0.010735 | 0.309993 | 0.0017576 | 1.00E-09 | 4.9295E-05 | 22.720323 |
|  | 5 | rs4318925 | T | C | -0.015038 | 0.177245 | 0.0021207 | 1.30E-12 | 6.5953E-05 | 30.398487 |
|  | 6 | rs6957745 | C | T | -0.012179 | 0.202978 | 0.0020233 | 1.80E-09 | 4.7993E-05 | 22.12007 |
|  | 7 | rs17317920 | G | A | 0.0090603 | 0.479238 | 0.0016312 | 2.80E-08 | 4.0974E-05 | 18.884631 |
|  | 8 | rs35287743 | T | G | -0.01774 | 0.11583 | 0.0025524 | 3.60E-12 | 6.4461E-05 | 29.710703 |
|  | 9 | rs7148387 | G | A | -0.009306 | 0.590715 | 0.0016502 | 1.70E-08 | 4.188E-05 | 19.302251 |
|  | 10 | rs56094641 | G | A | 0.0125875 | 0.404619 | 0.0016511 | 2.50E-14 | 7.634E-05 | 35.185955 |
|  | 11 | rs838133 | G | A | 0.016184 | 0.549409 | 0.0016763 | 4.70E-22 | 0.00012968 | 59.775376 |
| **Oily fish intake** | 1 | rs973526 | T | C | -0.011507 | 0.513307 | 0.0019296 | 2.50E-09 | 6.6159E-05 | 30.464162 |
|  | 2 | rs45501495 | T | C | 0.0156803 | 0.236013 | 0.0022571 | 3.70E-12 | 8.8667E-05 | 40.829412 |
|  | 3 | rs55930451 | T | C | -0.017054 | 0.108001 | 0.0030757 | 2.90E-08 | 5.6036E-05 | 25.802863 |
|  | 4 | rs55985303 | A | G | 0.0129737 | 0.241079 | 0.0022363 | 6.60E-09 | 6.159E-05 | 28.360533 |
|  | 5 | rs17050031 | T | C | -0.012049 | 0.4801 | 0.0019207 | 3.50E-10 | 7.2477E-05 | 33.373627 |
|  | 6 | rs275160 | C | T | 0.0121188 | 0.700597 | 0.002101 | 8.00E-09 | 6.1613E-05 | 28.370984 |
|  | 7 | rs13070166 | A | T | 0.0142105 | 0.228586 | 0.0022781 | 4.40E-10 | 7.1217E-05 | 32.793727 |
|  | 8 | rs114497213 | T | G | 0.0273225 | 0.05481 | 0.0042361 | 1.10E-10 | 7.7348E-05 | 35.617003 |
|  | 9 | rs10513136 | A | G | -0.023323 | 0.065424 | 0.0038631 | 1.60E-09 | 6.6521E-05 | 30.630978 |
|  | 10 | rs1876245 | C | T | 0.0151162 | 0.431482 | 0.0019312 | 5.00E-15 | 0.0001121 | 51.62319 |
|  | 11 | rs10510554 | C | T | 0.0110446 | 0.569277 | 0.0019364 | 1.20E-08 | 5.9821E-05 | 27.545563 |
|  | 12 | rs905575 | G | C | 0.0138824 | 0.82397 | 0.0025192 | 3.60E-08 | 5.5906E-05 | 25.742781 |
|  | 13 | rs9841174 | C | T | 0.0147781 | 0.373913 | 0.0019803 | 8.50E-14 | 0.00010225 | 47.085897 |
|  | 14 | rs1201289 | G | T | -0.010727 | 0.394525 | 0.0019595 | 4.40E-08 | 5.4969E-05 | 25.311313 |
|  | 15 | rs7683782 | G | C | 0.0144775 | 0.833405 | 0.002574 | 1.90E-08 | 5.8202E-05 | 26.79998 |
|  | 16 | rs10076975 | C | T | 0.0112394 | 0.381413 | 0.0019672 | 1.10E-08 | 5.9609E-05 | 27.448108 |
|  | 17 | rs10061973 | T | G | -0.010852 | 0.5139 | 0.001916 | 1.50E-08 | 5.8839E-05 | 27.093265 |
|  | 18 | rs16891727 | A | C | -0.023717 | 0.129814 | 0.00284 | 6.80E-17 | 0.00012708 | 58.522558 |
|  | 19 | rs34555420 | T | G | -0.023768 | 0.097821 | 0.0032191 | 1.50E-13 | 9.9707E-05 | 45.913749 |
|  | 20 | rs12663865 | A | G | 0.012772 | 0.758185 | 0.0022324 | 1.10E-08 | 5.9814E-05 | 27.542681 |
|  | 21 | rs4869859 | C | T | 0.0140098 | 0.449939 | 0.0019221 | 3.10E-13 | 9.7153E-05 | 44.737792 |
|  | 22 | rs11767283 | G | A | 0.0176735 | 0.221709 | 0.0023194 | 2.50E-14 | 0.0001078 | 49.638799 |
|  | 23 | rs6465487 | G | A | -0.012354 | 0.399814 | 0.0019563 | 2.70E-10 | 7.3241E-05 | 33.7256 |
|  | 24 | rs11986122 | G | C | 0.0148173 | 0.422568 | 0.0019494 | 2.90E-14 | 0.00010714 | 49.33852 |
|  | 25 | rs790564 | C | A | 0.0146906 | 0.722952 | 0.0021477 | 7.90E-12 | 8.6452E-05 | 39.80935 |
|  | 26 | rs552234 | A | G | -0.011647 | 0.495449 | 0.0019125 | 1.10E-09 | 6.7825E-05 | 31.231687 |
|  | 27 | rs9886779 | A | T | -0.010721 | 0.439213 | 0.0019288 | 2.70E-08 | 5.6616E-05 | 26.069931 |
|  | 28 | rs10828250 | G | C | -0.020146 | 0.309332 | 0.0020734 | 2.60E-22 | 0.00017342 | 79.865603 |
|  | 29 | rs703987 | C | G | 0.0111218 | 0.615005 | 0.0019736 | 1.70E-08 | 5.8575E-05 | 26.972011 |
|  | 30 | rs61882686 | A | C | 0.0197559 | 0.085234 | 0.0034254 | 8.00E-09 | 6.0862E-05 | 28.025085 |
|  | 31 | rs4278546 | G | A | 0.0125555 | 0.441085 | 0.0019381 | 9.30E-11 | 7.7726E-05 | 35.790999 |
|  | 32 | rs2374424 | G | A | -0.011444 | 0.601525 | 0.0019561 | 4.90E-09 | 6.2779E-05 | 28.908059 |
|  | 33 | rs510161 | G | C | -0.0113 | 0.31037 | 0.0020662 | 4.50E-08 | 5.466E-05 | 25.168951 |
|  | 34 | rs631490 | C | G | -0.01514 | 0.709106 | 0.0021025 | 6.00E-13 | 9.4562E-05 | 43.544294 |
|  | 35 | rs303817 | G | A | 0.0135799 | 0.751146 | 0.0022097 | 8.00E-10 | 6.8943E-05 | 31.746515 |
|  | 36 | rs35287743 | T | G | -0.02822 | 0.115869 | 0.0030107 | 7.00E-21 | 0.00016317 | 75.140802 |
|  | 37 | rs9597870 | G | T | -0.012745 | 0.245844 | 0.0022305 | 1.10E-08 | 6.0231E-05 | 27.734722 |
|  | 38 | rs3124402 | G | A | -0.022001 | 0.733261 | 0.0021565 | 1.90E-24 | 0.00018935 | 87.203109 |
|  | 39 | rs12855717 | T | C | -0.01223 | 0.526774 | 0.0019228 | 2.00E-10 | 7.457E-05 | 34.337448 |
|  | 40 | rs1361016 | G | T | 0.0149567 | 0.844853 | 0.002653 | 1.70E-08 | 5.8644E-05 | 27.003842 |
|  | 41 | rs9301837 | A | C | -0.015743 | 0.143273 | 0.0027305 | 8.10E-09 | 6.0842E-05 | 28.01568 |
|  | 42 | rs4982738 | A | G | 0.0108608 | 0.582899 | 0.0019694 | 3.50E-08 | 5.7357E-05 | 26.411135 |
|  | 43 | rs12896749 | C | G | -0.010964 | 0.384721 | 0.0019661 | 2.50E-08 | 5.6911E-05 | 26.205513 |
|  | 44 | rs1951286 | G | T | -0.014583 | 0.644911 | 0.0019995 | 3.00E-13 | 9.7404E-05 | 44.853353 |
|  | 45 | rs28533540 | A | G | 0.0146421 | 0.534203 | 0.0019248 | 2.80E-14 | 0.00010669 | 49.131505 |
|  | 46 | rs1421085 | C | T | 0.0184814 | 0.40341 | 0.0019487 | 2.50E-21 | 0.00016441 | 75.712529 |
|  | 47 | rs11859365 | C | A | 0.0225669 | 0.253765 | 0.0021968 | 9.40E-25 | 0.00019288 | 88.825754 |
|  | 48 | rs9889161 | T | G | -0.01332 | 0.357929 | 0.0020015 | 2.80E-11 | 8.155E-05 | 37.552107 |
|  | 49 | rs28623270 | T | A | -0.017833 | 0.14872 | 0.0027375 | 7.30E-11 | 8.0521E-05 | 37.078302 |
|  | 50 | rs2952140 | T | C | -0.010677 | 0.482607 | 0.001915 | 2.50E-08 | 5.6934E-05 | 26.216451 |
|  | 51 | rs4510068 | T | G | -0.013014 | 0.402798 | 0.0019708 | 4.00E-11 | 8.1475E-05 | 37.517696 |
|  | 52 | rs7243428 | G | A | -0.012978 | 0.224648 | 0.0022921 | 1.50E-08 | 5.8678E-05 | 27.019247 |
|  | 53 | rs9958909 | G | T | 0.0157617 | 0.139718 | 0.0027781 | 1.40E-08 | 5.9721E-05 | 27.499781 |
|  | 54 | rs59355765 | T | C | -0.016252 | 0.160079 | 0.0026097 | 4.70E-10 | 7.1028E-05 | 32.70634 |
|  | 55 | rs4002471 | T | C | -0.019244 | 0.547362 | 0.0019244 | 1.50E-23 | 0.0001835 | 84.507552 |
|  | 56 | rs7254235 | G | A | -0.01063 | 0.577254 | 0.0019401 | 4.30E-08 | 5.5154E-05 | 25.396485 |
|  | 57 | rs75887709 | G | A | -0.015911 | 0.13588 | 0.0028148 | 1.60E-08 | 5.9447E-05 | 27.373638 |
|  | 58 | rs12983532 | T | C | -0.01339 | 0.251137 | 0.0022333 | 2.00E-09 | 6.7433E-05 | 31.050962 |
|  | 59 | rs6033437 | A | C | 0.0124683 | 0.257329 | 0.0022098 | 1.70E-08 | 5.942E-05 | 27.360843 |
|  | 60 | rs6059844 | G | A | 0.0110001 | 0.495121 | 0.0019145 | 9.20E-09 | 6.0495E-05 | 27.85622 |
|  | 61 | rs6089753 | T | C | -0.011542 | 0.530967 | 0.0019184 | 1.80E-09 | 6.6353E-05 | 30.553845 |
|  | 62 | rs2827161 | G | T | 0.0107114 | 0.422842 | 0.0019372 | 3.20E-08 | 5.6001E-05 | 25.786572 |
|  | 63 | rs9606833 | C | T | 0.0169856 | 0.243593 | 0.0022309 | 2.70E-14 | 0.00010632 | 48.958972 |
| **Cereal intake** | 1 | rs10857964 | C | T | 0.0140866 | 0.205117 | 0.0022053 | 1.70E-10 | 6.4706E-05 | 28.578653 |
|  | 2 | rs12354267 | C | T | 0.0116473 | 0.309145 | 0.0019328 | 1.70E-09 | 5.7947E-05 | 25.593003 |
|  | 3 | rs112780312 | A | G | -0.012148 | 0.274969 | 0.0020189 | 1.80E-09 | 5.8844E-05 | 25.989224 |
|  | 4 | rs184643 | A | G | -0.012165 | 0.56672 | 0.0018047 | 1.60E-11 | 7.268E-05 | 32.100428 |
|  | 5 | rs6545770 | T | A | -0.013728 | 0.748101 | 0.0020604 | 2.70E-11 | 7.1028E-05 | 31.37101 |
|  | 6 | rs4988235 | A | G | 0.0114 | 0.736893 | 0.0020124 | 1.50E-08 | 5.0394E-05 | 22.256909 |
|  | 7 | rs67723420 | A | T | 0.0105252 | 0.376335 | 0.0018467 | 1.20E-08 | 5.2002E-05 | 22.967076 |
|  | 8 | rs7619139 | A | T | -0.016951 | 0.588533 | 0.001815 | 9.70E-21 | 0.00013916 | 61.468604 |
|  | 9 | rs9846396 | T | C | 0.0119674 | 0.441556 | 0.0018003 | 3.00E-11 | 7.0631E-05 | 31.195514 |
|  | 10 | rs11097340 | T | C | -0.011533 | 0.399592 | 0.001815 | 2.10E-10 | 6.3828E-05 | 28.190457 |
|  | 11 | rs3115230 | A | C | -0.011478 | 0.752001 | 0.0020713 | 3.00E-08 | 4.9139E-05 | 21.702561 |
|  | 12 | rs11940694 | G | A | -0.012665 | 0.604068 | 0.0018343 | 5.00E-12 | 7.6723E-05 | 33.886444 |
|  | 13 | rs10057775 | C | T | 0.0200293 | 0.893563 | 0.0028938 | 4.50E-12 | 7.631E-05 | 33.703804 |
|  | 14 | rs79642906 | A | G | -0.018158 | 0.083314 | 0.0032281 | 1.90E-08 | 5.0363E-05 | 22.243222 |
|  | 15 | rs1853931 | A | G | -0.011337 | 0.531294 | 0.0018102 | 3.80E-10 | 6.401E-05 | 28.270964 |
|  | 16 | rs6918737 | A | T | 0.0137325 | 0.234488 | 0.0021098 | 7.60E-11 | 6.7702E-05 | 29.901824 |
|  | 17 | rs2817377 | A | G | 0.0099008 | 0.537948 | 0.0017893 | 3.10E-08 | 4.8731E-05 | 21.522506 |
|  | 18 | rs2504706 | C | T | 0.0181793 | 0.234682 | 0.0021024 | 5.30E-18 | 0.00011872 | 52.435331 |
|  | 19 | rs9374896 | T | C | 0.0175265 | 0.466274 | 0.0017917 | 1.30E-22 | 0.00015289 | 67.532494 |
|  | 20 | rs4410790 | C | T | -0.010914 | 0.630668 | 0.001847 | 3.40E-09 | 5.5493E-05 | 24.509251 |
|  | 21 | rs62442924 | T | C | 0.0127309 | 0.194276 | 0.0022571 | 1.70E-08 | 5.074E-05 | 22.410014 |
|  | 22 | rs13234131 | G | A | 0.0170117 | 0.128369 | 0.0026602 | 1.60E-10 | 6.4762E-05 | 28.603081 |
|  | 23 | rs9987289 | G | A | 0.0178679 | 0.908767 | 0.0030947 | 7.80E-09 | 5.294E-05 | 23.381424 |
|  | 24 | rs4739095 | A | G | -0.012866 | 0.765732 | 0.0021052 | 9.90E-10 | 5.9385E-05 | 26.228417 |
|  | 25 | rs2927238 | G | T | 0.0102465 | 0.613181 | 0.0018288 | 2.10E-08 | 4.9806E-05 | 21.997111 |
|  | 26 | rs2799849 | T | C | -0.012329 | 0.678123 | 0.0019057 | 9.80E-11 | 6.6356E-05 | 29.307065 |
|  | 27 | rs7040561 | A | T | -0.016269 | 0.850635 | 0.00252 | 1.10E-10 | 6.7254E-05 | 29.703851 |
|  | 28 | rs491711 | C | A | 0.0116885 | 0.311975 | 0.0019337 | 1.50E-09 | 5.865E-05 | 25.9038 |
|  | 29 | rs2450126 | G | A | -0.014902 | 0.156746 | 0.0024567 | 1.30E-09 | 5.8704E-05 | 25.927467 |
|  | 30 | rs10837531 | G | C | 0.0107836 | 0.455002 | 0.0017969 | 2.00E-09 | 5.7672E-05 | 25.471659 |
|  | 31 | rs11038810 | G | A | 0.0111343 | 0.644091 | 0.0018633 | 2.30E-09 | 5.6838E-05 | 25.103435 |
|  | 32 | rs627185 | G | C | -0.010827 | 0.544427 | 0.0017908 | 1.50E-09 | 5.8148E-05 | 25.681925 |
|  | 33 | rs2472297 | T | C | -0.015852 | 0.261458 | 0.0020219 | 4.50E-15 | 9.7043E-05 | 42.862044 |
|  | 34 | rs1104608 | C | G | 0.0108629 | 0.426239 | 0.0018184 | 2.30E-09 | 5.7717E-05 | 25.49161 |
|  | 35 | rs68136852 | A | C | -0.014122 | 0.152389 | 0.0024785 | 1.20E-08 | 5.1521E-05 | 22.754837 |
|  | 36 | rs3859193 | A | T | -0.010326 | 0.470074 | 0.0017993 | 9.50E-09 | 5.312E-05 | 23.4611 |
|  | 37 | rs8097544 | G | A | -0.024637 | 0.145206 | 0.0025411 | 3.20E-22 | 0.00015068 | 66.554358 |
|  | 38 | rs4797242 | A | C | 0.0114312 | 0.297207 | 0.0019489 | 4.50E-09 | 5.4588E-05 | 24.109613 |
|  | 39 | rs11670024 | G | A | 0.0160114 | 0.115508 | 0.0027995 | 1.10E-08 | 5.2384E-05 | 23.13576 |
|  | 40 | rs6510177 | C | T | -0.013035 | 0.805589 | 0.0022874 | 1.20E-08 | 5.3225E-05 | 23.507294 |
|  | 41 | rs78854891 | C | T | 0.0221411 | 0.065728 | 0.0036331 | 1.10E-09 | 6.0208E-05 | 26.591615 |
|  | 42 | rs56131196 | A | G | 0.0180024 | 0.188724 | 0.002277 | 2.70E-15 | 9.924E-05 | 43.832486 |
|  | 43 | rs838133 | G | A | -0.020652 | 0.549201 | 0.0018433 | 3.90E-29 | 0.00021118 | 93.283669 |
| **Salted nuts intake** | 1 | rs116089899 | G | C | 0.0473616 | 0.007766 | 0.0094875 | 6E-07 | 3.457E-05 | 2.2452687 |
|  | 2 | rs55830729 | C | G | 0.0201115 | 0.037865 | 0.0042508 | 2.2E-06 | 2.9471E-05 | 1.9141007 |
|  | 3 | rs79900875 | G | A | 0.0190884 | 0.041978 | 0.0041157 | 3.5E-06 | 2.9307E-05 | 1.9034352 |
|  | 4 | rs4972701 | T | C | -0.007499 | 0.467924 | 0.0016186 | 3.6E-06 | 2.8004E-05 | 1.8188331 |
|  | 5 | rs144425541 | A | G | 0.0393736 | 0.011905 | 0.0081424 | 1.3E-06 | 3.6473E-05 | 2.3688812 |
|  | 6 | rs1653772 | G | A | -0.030843 | 0.984534 | 0.0065054 | 0.0000021 | 2.8971E-05 | 1.8816207 |
|  | 7 | rs146665115 | A | G | 0.0449273 | 0.008046 | 0.0097191 | 3.8E-06 | 3.222E-05 | 2.0926437 |
|  | 8 | rs55768166 | G | A | 0.0096239 | 0.181033 | 0.0020966 | 4.4E-06 | 2.7464E-05 | 1.783734 |
|  | 9 | rs80265037 | T | G | 0.0171419 | 0.049991 | 0.0037279 | 4.3E-06 | 2.791E-05 | 1.8127532 |
|  | 10 | rs13284665 | G | A | 0.0144441 | 0.131761 | 0.002376 | 1.2E-09 | 4.7735E-05 | 3.1003946 |
|  | 11 | rs10996983 | T | C | 0.0253131 | 0.021882 | 0.0055208 | 4.5E-06 | 2.7428E-05 | 1.7814348 |
|  | 12 | rs906822 | T | G | 0.0151811 | 0.067202 | 0.0032268 | 0.0000025 | 2.8894E-05 | 1.8766269 |
|  | 13 | rs10845793 | G | C | -0.013052 | 0.900684 | 0.0027232 | 0.0000016 | 3.0476E-05 | 1.9794057 |
|  | 14 | rs506454 | C | T | 0.0360269 | 0.011994 | 0.0076509 | 0.0000025 | 3.0761E-05 | 1.9979281 |
|  | 15 | rs147958200 | T | C | 0.0320672 | 0.015339 | 0.0068791 | 3.1E-06 | 3.1062E-05 | 2.0174763 |
|  | 16 | rs150856188 | T | G | 0.0394604 | 0.010508 | 0.0080489 | 9.5E-07 | 3.2381E-05 | 2.103093 |
|  | 17 | rs1381597 | T | C | -0.008274 | 0.716245 | 0.0017818 | 3.4E-06 | 2.7826E-05 | 1.8072465 |
|  | 18 | rs75342409 | C | T | 0.0354039 | 0.015501 | 0.0066638 | 1.1E-07 | 3.8257E-05 | 2.4847512 |
|  | 19 | rs138188349 | G | A | 0.0313199 | 0.017319 | 0.0063542 | 8.3E-07 | 3.3389E-05 | 2.1686012 |
|  | 20 | rs80261113 | T | G | 0.0537207 | 0.007293 | 0.0099123 | 6E-08 | 4.1787E-05 | 2.7140501 |
|  | 21 | rs117345122 | T | G | 0.0199755 | 0.039794 | 0.0041461 | 0.0000015 | 3.0494E-05 | 1.980522 |
|  | 22 | rs73085383 | T | C | 0.0372358 | 0.014743 | 0.0070522 | 1.3E-07 | 4.028E-05 | 2.6161543 |
|  | 23 | rs9613266 | C | T | -0.007755 | 0.468321 | 0.0016051 | 1.4E-06 | 2.9951E-05 | 1.9453011 |
| **Unsalted nuts intake** | 1 | rs73913809 | A | G | 0.0240023 | 0.064174 | 0.0052421 | 4.7E-06 | 6.9197E-05 | 4.4944761 |
|  | 2 | rs2664108 | G | A | -0.012813 | 0.690161 | 0.0027965 | 4.6E-06 | 7.0215E-05 | 4.5605928 |
|  | 3 | rs146575647 | C | T | 0.0788781 | 0.007249 | 0.0161265 | 0.000001 | 8.9549E-05 | 5.8164674 |
|  | 4 | rs140760808 | G | C | 0.0752483 | 0.007015 | 0.0162129 | 3.5E-06 | 7.8885E-05 | 5.1237402 |
|  | 5 | rs11241419 | C | G | 0.025021 | 0.068244 | 0.0050699 | 0.0000008 | 7.9617E-05 | 5.1712992 |
|  | 6 | rs4734720 | C | T | 0.0213049 | 0.084401 | 0.0045936 | 3.5E-06 | 7.0152E-05 | 4.5565009 |
|  | 7 | rs117713405 | A | T | 0.083463 | 0.006297 | 0.0170891 | 0.000001 | 8.7178E-05 | 5.6624611 |
|  | 8 | rs139312083 | C | T | 0.0525489 | 0.014654 | 0.010873 | 1.3E-06 | 7.9745E-05 | 5.1795965 |
|  | 9 | rs2178664 | C | T | 0.0315804 | 0.037859 | 0.0069024 | 4.8E-06 | 7.2656E-05 | 4.7191498 |
|  | 10 | rs76520208 | T | C | 0.0372704 | 0.028099 | 0.0077766 | 0.0000016 | 7.587E-05 | 4.9279131 |
|  | 11 | rs4904335 | G | A | -0.020568 | 0.092895 | 0.0044218 | 3.3E-06 | 7.1293E-05 | 4.6305992 |
|  | 12 | rs77536627 | T | C | 0.0356537 | 0.030014 | 0.0076356 | 3E-06 | 7.4017E-05 | 4.8075055 |
|  | 13 | rs62030847 | T | C | 0.0252204 | 0.057422 | 0.0054937 | 4.4E-06 | 6.8854E-05 | 4.4721725 |
|  | 14 | rs117987918 | A | G | 0.0514303 | 0.014655 | 0.0110851 | 3.5E-06 | 7.6391E-05 | 4.9617459 |
|  | 15 | rs112734217 | T | A | 0.032244 | 0.038438 | 0.0067555 | 1.8E-06 | 7.6854E-05 | 4.9918137 |
|  | 16 | rs145713169 | A | G | 0.0354878 | 0.030457 | 0.0075697 | 2.8E-06 | 7.4378E-05 | 4.8309634 |
| **Type of fat/oil used in cooking: Olive oil** | 1 | rs1152834 | C | T | -0.013077 | 0.587622 | 0.0027709 | 2.4E-06 | 8.2882E-05 | 5.383377 |
|  | 2 | rs2161277 | T | C | -0.021193 | 0.098237 | 0.004446 | 1.9E-06 | 7.9574E-05 | 5.1684906 |
|  | 3 | rs6966854 | G | A | 0.034646 | 0.040778 | 0.0066834 | 2.2E-07 | 9.3903E-05 | 6.0993159 |
|  | 4 | rs6965810 | C | T | 0.0133768 | 0.67165 | 0.0028252 | 2.2E-06 | 7.8925E-05 | 5.1263455 |
|  | 5 | rs11137009 | A | C | -0.013511 | 0.44701 | 0.0027282 | 7.3E-07 | 9.0251E-05 | 5.8620653 |
|  | 6 | rs10970249 | T | C | 0.0247353 | 0.066244 | 0.0053397 | 3.6E-06 | 7.5691E-05 | 4.9162763 |
|  | 7 | rs3004203 | G | A | -0.026674 | 0.945934 | 0.0058328 | 4.8E-06 | 7.2775E-05 | 4.7268906 |
|  | 8 | rs7128860 | T | G | -0.015359 | 0.250165 | 0.0031805 | 1.4E-06 | 8.8496E-05 | 5.7480832 |
| Notes: SNP=single nucleotide polymorphism; EA=effect allele; OA=other allele; EAF=effect allele frequency | | | | | | | | |  |  |

**Supplemental Table 2.** **The results of Cochran's Q test and MR-Egger intercept test.**

| **Exposure** | **Outcome** | **MR Method** | **MR Egger Intercept** | **Intercept SE** | **Intercept P** | **Q** | **Q_df** | **Q_pval** |
| --- | --- | --- | --- | --- | --- | --- | --- | --- |
| **Fresh fruit intake** | **Overall breast cancer** | Inverse variance weighted | |  |  | 136.43685 | 51 | 1.01E-09 |
|  |  | MR Egger | 0.005260698 | 0.005448384 | 0.33891551 | 133.93944 | 50 | 1.35E-09 |
|  | **ER+ Breast cancer** | Inverse variance weighted | |  |  | 126.01676 | 52 | 4.39E-08 |
|  |  | MR Egger | 0.00894378 | 0.006054889 | 0.145793153 | 120.84671 | 51 | 1.34E-07 |
|  | **ER- Breast cancer** | Inverse variance weighted | |  |  | 126.04388 | 52 | 4.36E-08 |
|  |  | MR Egger | 0.004257813 | 0.009420359 | 0.65319993 | 125.54101 | 51 | 3.18E-08 |
| **Dried fruit intake** | **Overall breast cancer** | Inverse variance weighted | |  |  | 108.01345 | 39 | 2.13E-08 |
|  |  | MR Egger | 0.001067309 | 0.008476347 | 0.900462163 | 107.9684 | 38 | 1.26E-08 |
|  | **ER+ Breast cancer** | Inverse variance weighted | |  |  | 116.39197 | 39 | 1.24E-09 |
|  |  | MR Egger | 0.004634604 | 0.010453335 | 0.660018496 | 115.79298 | 38 | 8.60E-10 |
|  | **ER- Breast cancer** | Inverse variance weighted | |  |  | 54.488615 | 39 | 0.0507871 |
|  |  | MR Egger | -0.011515283 | 0.010813482 | 0.293640908 | 52.909661 | 38 | 0.0546533 |
| **Cooked vegetable intake** | **Overall breast cancer** | Inverse variance weighted | |  |  | 117.97282 | 16 | 1.35E-17 |
|  |  | MR Egger | 0.020324008 | 0.050854444 | 0.695046829 | 116.72988 | 15 | 8.14E-18 |
|  | **ER+ Breast cancer** | Inverse variance weighted | |  |  | 79.782408 | 16 | 1.82E-10 |
|  |  | MR Egger | 0.024760997 | 0.050031472 | 0.627835281 | 78.500575 | 15 | 1.31E-10 |
|  | **ER- Breast cancer** | Inverse variance weighted | |  |  | 59.969255 | 16 | 5.30E-07 |
|  |  | MR Egger | -0.029974926 | 0.066125591 | 0.656819134 | 59.158844 | 15 | 3.52E-07 |
| **Salad / raw vegetable intake** | **Overall breast cancer** | Inverse variance weighted | |  |  | 82.60679 | 17 | 1.32E-10 |
|  |  | MR Egger | 0.021737217 | 0.020388771 | 0.302186094 | 77.127609 | 16 | 5.46E-10 |
|  | **ER+ Breast cancer** | Inverse variance weighted | |  |  | 81.243648 | 17 | 2.30E-10 |
|  |  | MR Egger | 0.019829023 | 0.024437661 | 0.429035132 | 78.032652 | 16 | 3.76E-10 |
|  | **ER- Breast cancer** | Inverse variance weighted | |  |  | 19.352536 | 17 | 0.3086762 |
|  |  | MR Egger | 0.022419181 | 0.017643698 | 0.222014422 | 17.578649 | 16 | 0.3491406 |
| **Non-oily fish intake** | **Overall breast cancer** | Inverse variance weighted | |  |  | 94.065499 | 9 | 2.48E-16 |
|  |  | MR Egger | 0.014574703 | 0.038497277 | 0.714841408 | 92.409852 | 8 | 1.51E-16 |
|  | **ER+ Breast cancer** | Inverse variance weighted | |  |  | 59.341008 | 9 | 1.80E-09 |
|  |  | MR Egger | 0.012174191 | 0.036563066 | 0.74771933 | 58.529893 | 8 | 9.05E-10 |
|  | **ER- Breast cancer** | Inverse variance weighted | |  |  | 45.403455 | 9 | 7.77E-07 |
|  |  | MR Egger | 0.013559405 | 0.049045259 | 0.789197544 | 44.973764 | 8 | 3.72E-07 |
| **Oily fish intake** | **Overall breast cancer** | Inverse variance weighted | |  |  | 269.36085 | 57 | 2.54E-29 |
|  |  | MR Egger | 0.004163832 | 0.009149097 | 0.650790733 | 268.36825 | 56 | 1.71E-29 |
|  | **ER+ Breast cancer** | Inverse variance weighted | |  |  | 225.03275 | 57 | 8.03E-22 |
|  |  | MR Egger | 0.005674019 | 0.009983302 | 0.572068823 | 223.74215 | 56 | 6.48E-22 |
|  | **ER- Breast cancer** | Inverse variance weighted | |  |  | 151.48549 | 57 | 1.66E-10 |
|  |  | MR Egger | -0.007331831 | 0.01248224 | 0.559308833 | 150.5579 | 56 | 1.34E-10 |
| **Cereal intake** | **Overall breast cancer** | Inverse variance weighted | |  |  | 130.56853 | 38 | 4.41E-12 |
|  |  | MR Egger | 0.010740107 | 0.009523049 | 0.266664362 | 126.22919 | 37 | 1.13E-11 |
|  | **ER+ Breast cancer** | Inverse variance weighted | |  |  | 113.60903 | 38 | 1.83E-09 |
|  |  | MR Egger | 0.010229349 | 0.010640623 | 0.342617708 | 110.84044 | 37 | 2.69E-09 |
|  | **ER- Breast cancer** | Inverse variance weighted | |  |  | 42.466858 | 38 | 0.284533 |
|  |  | MR Egger | 0.006918294 | 0.009996829 | 0.493227054 | 41.924187 | 37 | 0.2657846 |
| **Salted nuts intake** | **Overall breast cancer** | Inverse variance weighted | |  |  | 11.858741 | 21 | 0.943353 |
|  |  | MR Egger | 0.006710925 | 0.005236297 | 0.214636835 | 10.2162 | 20 | 0.96408 |
|  | **ER+ Breast cancer** | Inverse variance weighted | |  |  | 22.305602 | 21 | 0.3820865 |
|  |  | MR Egger | 0.001192117 | 0.006559067 | 0.857607561 | 22.268821 | 20 | 0.3261037 |
|  | **ER- Breast cancer** | Inverse variance weighted | |  |  | 27.548926 | 21 | 0.1534139 |
|  |  | MR Egger | 0.019329933 | 0.010367498 | 0.07699978 | 23.4696 | 20 | 0.2663285 |
| **Unsalted nuts intake** | **Overall breast cancer** | Inverse variance weighted | |  |  | 18.897923 | 14 | 0.1688864 |
|  |  | MR Egger | -0.012638091 | 0.009010309 | 0.184156379 | 16.413918 | 13 | 0.2274988 |
|  | **ER+ Breast cancer** | Inverse variance weighted | |  |  | 26.783343 | 14 | 0.0205405 |
|  |  | MR Egger | -0.021958207 | 0.012158679 | 0.094119336 | 21.411486 | 13 | 0.0651785 |
|  | **ER- Breast cancer** | Inverse variance weighted | |  |  | 16.149214 | 14 | 0.3043472 |
|  |  | MR Egger | -0.004640528 | 0.016259246 | 0.779827784 | 16.048653 | 13 | 0.2465126 |
| **Type of fat/oil used in cooking: Olive oil** | **Overall breast cancer** | Inverse variance weighted | |  |  | 9.0335438 | 7 | 0.2502567 |
|  |  | MR Egger | 0.020375797 | 0.010337626 | 0.096212413 | 5.1485744 | 6 | 0.5249036 |
|  | **ER+ Breast cancer** | Inverse variance weighted | |  |  | 8.694202 | 7 | 0.275363 |
|  |  | MR Egger | 0.021209962 | 0.01236237 | 0.13703846 | 5.7506186 | 6 | 0.4516978 |
|  | **ER- Breast cancer** | Inverse variance weighted | |  |  | 1.4258398 | 7 | 0.9847666 |
|  |  | MR Egger | -0.001438576 | 0.01874779 | 0.941330601 | 1.4199518 | 6 | 0.9646335 |

**Supplemental Table 3. Causal effects of 7 dietary habits on overall BC risks using MR-Egger, weighted median, inverse variance weighted, simple mode, and weighted mode methods**

| **Outcome** | **Exposure** | **Method** | **nsnp** | **b** | **se** | **pval** | **or** | **or_lci95** | **or_uci95** |
| --- | --- | --- | --- | --- | --- | --- | --- | --- | --- |
| Breast cancer (Combined Oncoarray; iCOGS; GWAS meta analysis) \|\| id:ieu-a-1126 | Fresh fruit intake \|\| id:ukb-b-3881 | MR Egger | 48 | -0.451157 | 0.4843318 | 0.3564565 | 0.636891 | 0.2464867 | 1.6456468 |
| Breast cancer (Combined Oncoarray; iCOGS; GWAS meta analysis) \|\| id:ieu-a-1126 | Fresh fruit intake \|\| id:ukb-b-3881 | Weighted median | 48 | -0.310478 | 0.1743349 | 0.0749245 | 0.7330967 | 0.520912 | 1.0317112 |
| Breast cancer (Combined Oncoarray; iCOGS; GWAS meta analysis) \|\| id:ieu-a-1126 | Fresh fruit intake \|\| id:ukb-b-3881 | Inverse variance weighted | 48 | -0.267296 | 0.1405745 | 0.0572422 | 0.7654462 | 0.5811058 | 1.0082636 |
| Breast cancer (Combined Oncoarray; iCOGS; GWAS meta analysis) \|\| id:ieu-a-1126 | Fresh fruit intake \|\| id:ukb-b-3881 | Simple mode | 48 | -0.332351 | 0.3960602 | 0.4056372 | 0.7172355 | 0.3300111 | 1.5588166 |
| Breast cancer (Combined Oncoarray; iCOGS; GWAS meta analysis) \|\| id:ieu-a-1126 | Fresh fruit intake \|\| id:ukb-b-3881 | Weighted mode | 48 | -0.332351 | 0.2788973 | 0.239377 | 0.7172355 | 0.4152022 | 1.238979 |
| **Outcome** | **Exposure** | **Method** | **nsnp** | **b** | **se** | **pval** | **or** | **or_lci95** | **or_uci95** |
| Breast cancer (Combined Oncoarray; iCOGS; GWAS meta analysis) \|\| id:ieu-a-1126 | Dried fruit intake \|\| id:ukb-b-16576 | MR Egger | 38 | -0.75054 | 0.599105 | 0.2183669 | 0.4721117 | 0.1459071 | 1.5276127 |
| Breast cancer (Combined Oncoarray; iCOGS; GWAS meta analysis) \|\| id:ieu-a-1126 | Dried fruit intake \|\| id:ukb-b-16576 | Weighted median | 38 | -0.525392 | 0.1544139 | 0.0006677 | 0.5913237 | 0.4369036 | 0.8003225 |
| Breast cancer (Combined Oncoarray; iCOGS; GWAS meta analysis) \|\| id:ieu-a-1126 | Dried fruit intake \|\| id:ukb-b-16576 | Inverse variance weighted | 38 | -0.600238 | 0.1255762 | 1.75E-06 | 0.548681 | 0.4289704 | 0.7017987 |
| Breast cancer (Combined Oncoarray; iCOGS; GWAS meta analysis) \|\| id:ieu-a-1126 | Dried fruit intake \|\| id:ukb-b-16576 | Simple mode | 38 | -0.510909 | 0.3553524 | 0.1589084 | 0.5999499 | 0.2989737 | 1.2039183 |
| Breast cancer (Combined Oncoarray; iCOGS; GWAS meta analysis) \|\| id:ieu-a-1126 | Dried fruit intake \|\| id:ukb-b-16576 | Weighted mode | 38 | -0.479174 | 0.3432997 | 0.1710983 | 0.6192948 | 0.3159911 | 1.2137243 |
| **Outcome** | **Exposure** | **Method** | **nsnp** | **b** | **se** | **pval** | **or** | **or_lci95** | **or_uci95** |
| Breast cancer (Combined Oncoarray; iCOGS; GWAS meta analysis) \|\| id:ieu-a-1126 | Cooked vegetable intake \|\| id:ukb-b-8089 | MR Egger | 17 | -2.371621 | 4.9577303 | 0.6392856 | 0.0933293 | 5.62E-06 | 1549.2559 |
| Breast cancer (Combined Oncoarray; iCOGS; GWAS meta analysis) \|\| id:ieu-a-1126 | Cooked vegetable intake \|\| id:ukb-b-8089 | Weighted median | 17 | -0.064229 | 0.2438927 | 0.7922803 | 0.9377899 | 0.5814333 | 1.5125551 |
| Breast cancer (Combined Oncoarray; iCOGS; GWAS meta analysis) \|\| id:ieu-a-1126 | Cooked vegetable intake \|\| id:ukb-b-8089 | Inverse variance weighted | 17 | -0.398464 | 0.4386554 | 0.3636796 | 0.6713504 | 0.2841569 | 1.5861355 |
| Breast cancer (Combined Oncoarray; iCOGS; GWAS meta analysis) \|\| id:ieu-a-1126 | Cooked vegetable intake \|\| id:ukb-b-8089 | Simple mode | 17 | -0.075451 | 0.3964723 | 0.8514631 | 0.9273252 | 0.426332 | 2.0170475 |
| Breast cancer (Combined Oncoarray; iCOGS; GWAS meta analysis) \|\| id:ieu-a-1126 | Cooked vegetable intake \|\| id:ukb-b-8089 | Weighted mode | 17 | -0.132281 | 0.3598828 | 0.7180133 | 0.8760949 | 0.4327257 | 1.7737385 |
| **Outcome** | **Exposure** | **Method** | **nsnp** | **b** | **se** | **pval** | **or** | **or_lci95** | **or_uci95** |
| Breast cancer (Combined Oncoarray; iCOGS; GWAS meta analysis) \|\| id:ieu-a-1126 | Salad / raw vegetable intake \|\| id:ukb-b-1996 | MR Egger | 16 | -0.438816 | 1.1843971 | 0.7165576 | 0.6447997 | 0.063277 | 6.5705842 |
| Breast cancer (Combined Oncoarray; iCOGS; GWAS meta analysis) \|\| id:ieu-a-1126 | Salad / raw vegetable intake \|\| id:ukb-b-1996 | Weighted median | 16 | 0.0353941 | 0.2765553 | 0.8981631 | 1.036028 | 0.6025081 | 1.7814763 |
| Breast cancer (Combined Oncoarray; iCOGS; GWAS meta analysis) \|\| id:ieu-a-1126 | Salad / raw vegetable intake \|\| id:ukb-b-1996 | Inverse variance weighted | 16 | 0.0810727 | 0.2259756 | 0.7197689 | 1.0844498 | 0.6963942 | 1.6887437 |
| Breast cancer (Combined Oncoarray; iCOGS; GWAS meta analysis) \|\| id:ieu-a-1126 | Salad / raw vegetable intake \|\| id:ukb-b-1996 | Simple mode | 16 | 0.0502919 | 0.5371407 | 0.9266431 | 1.051578 | 0.3669594 | 3.0134567 |
| Breast cancer (Combined Oncoarray; iCOGS; GWAS meta analysis) \|\| id:ieu-a-1126 | Salad / raw vegetable intake \|\| id:ukb-b-1996 | Weighted mode | 16 | -0.057065 | 0.4862634 | 0.9081357 | 0.9445323 | 0.3641674 | 2.4498109 |
| **Outcome** | **Exposure** | **Method** | **nsnp** | **b** | **se** | **pval** | **or** | **or_lci95** | **or_uci95** |
| Breast cancer (Combined Oncoarray; iCOGS; GWAS meta analysis) \|\| id:ieu-a-1126 | Non-oily fish intake \|\| id:ukb-b-17627 | MR Egger | 8 | 0.0522428 | 1.4783644 | 0.9729564 | 1.0536315 | 0.0581138 | 19.102842 |
| Breast cancer (Combined Oncoarray; iCOGS; GWAS meta analysis) \|\| id:ieu-a-1126 | Non-oily fish intake \|\| id:ukb-b-17627 | Weighted median | 8 | -0.114692 | 0.2784479 | 0.6804143 | 0.8916406 | 0.5166189 | 1.5388967 |
| Breast cancer (Combined Oncoarray; iCOGS; GWAS meta analysis) \|\| id:ieu-a-1126 | Non-oily fish intake \|\| id:ukb-b-17627 | Inverse variance weighted | 8 | -0.337613 | 0.2775281 | 0.2237951 | 0.7134717 | 0.4141332 | 1.2291742 |
| Breast cancer (Combined Oncoarray; iCOGS; GWAS meta analysis) \|\| id:ieu-a-1126 | Non-oily fish intake \|\| id:ukb-b-17627 | Simple mode | 8 | -0.003204 | 0.3479959 | 0.9929118 | 0.9968015 | 0.5039514 | 1.9716452 |
| Breast cancer (Combined Oncoarray; iCOGS; GWAS meta analysis) \|\| id:ieu-a-1126 | Non-oily fish intake \|\| id:ukb-b-17627 | Weighted mode | 8 | -0.082509 | 0.3241626 | 0.8064016 | 0.920803 | 0.4877912 | 1.7381993 |
| **Outcome** | **Exposure** | **Method** | **nsnp** | **b** | **se** | **pval** | **or** | **or_lci95** | **or_uci95** |
| Breast cancer (Combined Oncoarray; iCOGS; GWAS meta analysis) \|\| id:ieu-a-1126 | Oily fish intake \|\| id:ukb-b-2209 | MR Egger | 53 | 0.148836 | 0.4355961 | 0.7339931 | 1.1604826 | 0.494142 | 2.7253701 |
| Breast cancer (Combined Oncoarray; iCOGS; GWAS meta analysis) \|\| id:ieu-a-1126 | Oily fish intake \|\| id:ukb-b-2209 | Weighted median | 53 | -0.151316 | 0.1107819 | 0.1719743 | 0.8595764 | 0.6918072 | 1.0680312 |
| Breast cancer (Combined Oncoarray; iCOGS; GWAS meta analysis) \|\| id:ieu-a-1126 | Oily fish intake \|\| id:ukb-b-2209 | Inverse variance weighted | 53 | -0.159264 | 0.0943018 | 0.0912434 | 0.8527713 | 0.7088613 | 1.0258972 |
| Breast cancer (Combined Oncoarray; iCOGS; GWAS meta analysis) \|\| id:ieu-a-1126 | Oily fish intake \|\| id:ukb-b-2209 | Simple mode | 53 | -0.438966 | 0.3016148 | 0.1515733 | 0.6447028 | 0.3569602 | 1.164392 |
| Breast cancer (Combined Oncoarray; iCOGS; GWAS meta analysis) \|\| id:ieu-a-1126 | Oily fish intake \|\| id:ukb-b-2209 | Weighted mode | 53 | -0.419246 | 0.2994849 | 0.1674838 | 0.6575421 | 0.3655922 | 1.1826337 |
| **Outcome** | **Exposure** | **Method** | **nsnp** | **b** | **se** | **pval** | **or** | **or_lci95** | **or_uci95** |
| Breast cancer (Combined Oncoarray; iCOGS; GWAS meta analysis) \|\| id:ieu-a-1126 | Cereal intake \|\| id:ukb-b-15926 | MR Egger | 37 | -0.119206 | 0.5004695 | 0.8131246 | 0.8876253 | 0.3328292 | 2.3672161 |
| Breast cancer (Combined Oncoarray; iCOGS; GWAS meta analysis) \|\| id:ieu-a-1126 | Cereal intake \|\| id:ukb-b-15926 | Weighted median | 37 | -0.009099 | 0.1349586 | 0.9462482 | 0.9909425 | 0.7606228 | 1.2910039 |
| Breast cancer (Combined Oncoarray; iCOGS; GWAS meta analysis) \|\| id:ieu-a-1126 | Cereal intake \|\| id:ukb-b-15926 | Inverse variance weighted | 37 | 0.0239949 | 0.1162772 | 0.8365104 | 1.024285 | 0.8155371 | 1.2864649 |
| Breast cancer (Combined Oncoarray; iCOGS; GWAS meta analysis) \|\| id:ieu-a-1126 | Cereal intake \|\| id:ukb-b-15926 | Simple mode | 37 | -0.01139 | 0.2710421 | 0.9667119 | 0.9886744 | 0.5812161 | 1.6817792 |
| Breast cancer (Combined Oncoarray; iCOGS; GWAS meta analysis) \|\| id:ieu-a-1126 | Cereal intake \|\| id:ukb-b-15926 | Weighted mode | 37 | -0.001329 | 0.21628 | 0.9951325 | 0.9986723 | 0.6536146 | 1.5258936 |
| **Outcome** | **Exposure** | **Method** | **nsnp** | **b** | **se** | **pval** | **or** | **or_lci95** | **or_uci95** |
| Breast cancer (Combined Oncoarray; iCOGS; GWAS meta analysis) \|\| id:ieu-a-1126 | Salted nuts intake | MR Egger | 22 | -0.120288 | 0.3203843 | 0.7112789 | 0.886665 | 0.4731981 | 1.6614076 |
| Breast cancer (Combined Oncoarray; iCOGS; GWAS meta analysis) \|\| id:ieu-a-1126 | Salted nuts intake | Weighted median | 22 | 0.0389743 | 0.2481356 | 0.8751908 | 1.0397438 | 0.6393064 | 1.6910001 |
| Breast cancer (Combined Oncoarray; iCOGS; GWAS meta analysis) \|\| id:ieu-a-1126 | Salted nuts intake | Inverse variance weighted | 22 | 0.2200323 | 0.1792615 | 0.2196581 | 1.246117 | 0.876937 | 1.7707173 |
| Breast cancer (Combined Oncoarray; iCOGS; GWAS meta analysis) \|\| id:ieu-a-1126 | Salted nuts intake | Simple mode | 22 | -0.029262 | 0.3947968 | 0.9416178 | 0.9711623 | 0.4479546 | 2.1054729 |
| Breast cancer (Combined Oncoarray; iCOGS; GWAS meta analysis) \|\| id:ieu-a-1126 | Salted nuts intake | Weighted mode | 22 | -0.029262 | 0.3726938 | 0.9381626 | 0.9711623 | 0.4677873 | 2.0162074 |
| **Outcome** | **Exposure** | **Method** | **nsnp** | **b** | **se** | **pval** | **or** | **or_lci95** | **or_uci95** |
| Breast cancer (Combined Oncoarray; iCOGS; GWAS meta analysis) \|\| id:ieu-a-1126 | Unsalted nuts intake | MR Egger | 15 | 0.4490868 | 0.3325178 | 0.1998772 | 1.5668806 | 0.8165655 | 3.0066353 |
| Breast cancer (Combined Oncoarray; iCOGS; GWAS meta analysis) \|\| id:ieu-a-1126 | Unsalted nuts intake | Weighted median | 15 | -0.037959 | 0.2164158 | 0.8607675 | 0.9627527 | 0.6299381 | 1.4714028 |
| Breast cancer (Combined Oncoarray; iCOGS; GWAS meta analysis) \|\| id:ieu-a-1126 | Unsalted nuts intake | Inverse variance weighted | 15 | 0.0427768 | 0.1688088 | 0.7999563 | 1.0437049 | 0.7496952 | 1.453017 |
| Breast cancer (Combined Oncoarray; iCOGS; GWAS meta analysis) \|\| id:ieu-a-1126 | Unsalted nuts intake | Simple mode | 15 | 0.6493577 | 0.4242718 | 0.1481661 | 1.9143109 | 0.8334221 | 4.3970351 |
| Breast cancer (Combined Oncoarray; iCOGS; GWAS meta analysis) \|\| id:ieu-a-1126 | Unsalted nuts intake | Weighted mode | 15 | -0.274016 | 0.3805992 | 0.483398 | 0.7603202 | 0.3605985 | 1.6031315 |
| **Outcome** | **Exposure** | **Method** | **nsnp** | **b** | **se** | **pval** | **or** | **or_lci95** | **or_uci95** |
| Breast cancer (Combined Oncoarray; iCOGS; GWAS meta analysis) \|\| id:ieu-a-1126 | Type of fat/oil used in cooking: Olive oil | MR Egger | 8 | -1.459094 | 0.6000789 | 0.051058 | 0.2324468 | 0.0717012 | 0.7535656 |
| Breast cancer (Combined Oncoarray; iCOGS; GWAS meta analysis) \|\| id:ieu-a-1126 | Type of fat/oil used in cooking: Olive oil | Weighted median | 8 | -0.401563 | 0.2414155 | 0.0962386 | 0.6692731 | 0.4169715 | 1.0742377 |
| Breast cancer (Combined Oncoarray; iCOGS; GWAS meta analysis) \|\| id:ieu-a-1126 | Type of fat/oil used in cooking: Olive oil | Inverse variance weighted | 8 | -0.333527 | 0.2094434 | 0.1112845 | 0.7163923 | 0.475192 | 1.0800223 |
| Breast cancer (Combined Oncoarray; iCOGS; GWAS meta analysis) \|\| id:ieu-a-1126 | Type of fat/oil used in cooking: Olive oil | Simple mode | 8 | -0.436883 | 0.4176386 | 0.3302959 | 0.646047 | 0.2849463 | 1.4647558 |
| Breast cancer (Combined Oncoarray; iCOGS; GWAS meta analysis) \|\| id:ieu-a-1126 | Type of fat/oil used in cooking: Olive oil | Weighted mode | 8 | -0.443753 | 0.3734329 | 0.2734608 | 0.6416241 | 0.3086087 | 1.3339918 |
| Notes: or = odds ratio; or_lci95 =the lower limit of 95% confidence interval; or_uci95= the upper limit of 95% confidence interval | | | | | | | | | |

**Supplemental Table 4.** **Causal effects of 7 dietary habits on ER+ BC risks using MR-Egger, weighted median, inverse variance weighted, simple mode, and weighted mode methods**

| **Outcome** | **Exposure** | **Method** | **nsnp** | **b** | **se** | **pval** | **or** | **or_lci95** | **or_uci95** |
| --- | --- | --- | --- | --- | --- | --- | --- | --- | --- |
| ER+ Breast cancer (Combined Oncoarray; iCOGS; GWAS meta analysis) \|\| id:ieu-a-1127 | Fresh fruit intake \|\| id:ukb-b-3881 | MR Egger | 51 | -1.061456 | 0.5198652 | 0.0465749 | 0.3459519 | 0.1248813 | 0.9583716 |
| ER+ Breast cancer (Combined Oncoarray; iCOGS; GWAS meta analysis) \|\| id:ieu-a-1127 | Fresh fruit intake \|\| id:ukb-b-3881 | Weighted median | 51 | -0.389123 | 0.204077 | 0.0565532 | 0.6776506 | 0.454247 | 1.0109267 |
| ER+ Breast cancer (Combined Oncoarray; iCOGS; GWAS meta analysis) \|\| id:ieu-a-1127 | Fresh fruit intake \|\| id:ukb-b-3881 | Inverse variance weighted | 51 | -0.218262 | 0.155239 | 0.1597315 | 0.8039147 | 0.593018 | 1.0898131 |
| ER+ Breast cancer (Combined Oncoarray; iCOGS; GWAS meta analysis) \|\| id:ieu-a-1127 | Fresh fruit intake \|\| id:ukb-b-3881 | Simple mode | 51 | -0.423001 | 0.4248382 | 0.3242027 | 0.6550783 | 0.2848811 | 1.5063392 |
| ER+ Breast cancer (Combined Oncoarray; iCOGS; GWAS meta analysis) \|\| id:ieu-a-1127 | Fresh fruit intake \|\| id:ukb-b-3881 | Weighted mode | 51 | -0.423001 | 0.3188069 | 0.1905949 | 0.6550783 | 0.3506867 | 1.2236779 |
| **Outcome** | **Exposure** | **Method** | **nsnp** | **b** | **se** | **pval** | **or** | **or_lci95** | **or_uci95** |
| ER+ Breast cancer (Combined Oncoarray; iCOGS; GWAS meta analysis) \|\| id:ieu-a-1127 | Dried fruit intake \|\| id:ukb-b-16576 | MR Egger | 38 | -0.627133 | 0.6822725 | 0.364118 | 0.5341208 | 0.1402418 | 2.034237 |
| ER+ Breast cancer (Combined Oncoarray; iCOGS; GWAS meta analysis) \|\| id:ieu-a-1127 | Dried fruit intake \|\| id:ukb-b-16576 | Weighted median | 38 | -0.295467 | 0.1769531 | 0.0949691 | 0.7441838 | 0.5260835 | 1.0527027 |
| ER+ Breast cancer (Combined Oncoarray; iCOGS; GWAS meta analysis) \|\| id:ieu-a-1127 | Dried fruit intake \|\| id:ukb-b-16576 | Inverse variance weighted | 38 | -0.476 | 0.1433151 | 0.0008958 | 0.6212633 | 0.4691195 | 0.8227501 |
| ER+ Breast cancer (Combined Oncoarray; iCOGS; GWAS meta analysis) \|\| id:ieu-a-1127 | Dried fruit intake \|\| id:ukb-b-16576 | Simple mode | 38 | -0.068792 | 0.4326165 | 0.8745223 | 0.9335206 | 0.3998281 | 2.1795883 |
| ER+ Breast cancer (Combined Oncoarray; iCOGS; GWAS meta analysis) \|\| id:ieu-a-1127 | Dried fruit intake \|\| id:ukb-b-16576 | Weighted mode | 38 | -0.017841 | 0.3969417 | 0.9643921 | 0.9823173 | 0.4511991 | 2.1386287 |
| **Outcome** | **Exposure** | **Method** | **nsnp** | **b** | **se** | **pval** | **or** | **or_lci95** | **or_uci95** |
| ER+ Breast cancer (Combined Oncoarray; iCOGS; GWAS meta analysis) \|\| id:ieu-a-1127 | Cooked vegetable intake \|\| id:ukb-b-8089 | MR Egger | 17 | -2.758714 | 4.8772861 | 0.5800121 | 0.0633732 | 4.47E-06 | 898.53558 |
| ER+ Breast cancer (Combined Oncoarray; iCOGS; GWAS meta analysis) \|\| id:ieu-a-1127 | Cooked vegetable intake \|\| id:ukb-b-8089 | Weighted median | 17 | -0.03884 | 0.2917133 | 0.8940804 | 0.961905 | 0.5430263 | 1.7038978 |
| ER+ Breast cancer (Combined Oncoarray; iCOGS; GWAS meta analysis) \|\| id:ieu-a-1127 | Cooked vegetable intake \|\| id:ukb-b-8089 | Inverse variance weighted | 17 | -0.354844 | 0.4316061 | 0.4109924 | 0.7012826 | 0.3009556 | 1.6341188 |
| ER+ Breast cancer (Combined Oncoarray; iCOGS; GWAS meta analysis) \|\| id:ieu-a-1127 | Cooked vegetable intake \|\| id:ukb-b-8089 | Simple mode | 17 | -0.011034 | 0.4593618 | 0.9811334 | 0.9890266 | 0.4019678 | 2.4334624 |
| ER+ Breast cancer (Combined Oncoarray; iCOGS; GWAS meta analysis) \|\| id:ieu-a-1127 | Cooked vegetable intake \|\| id:ukb-b-8089 | Weighted mode | 17 | -0.011034 | 0.4174344 | 0.9792388 | 0.9890266 | 0.4363959 | 2.2414823 |
| **Outcome** | **Exposure** | **Method** | **nsnp** | **b** | **se** | **pval** | **or** | **or_lci95** | **or_uci95** |
| ER+ Breast cancer (Combined Oncoarray; iCOGS; GWAS meta analysis) \|\| id:ieu-a-1127 | Salad / raw vegetable intake \|\| id:ukb-b-1996 | MR Egger | 16 | -0.176076 | 1.3000247 | 0.8941918 | 0.8385544 | 0.0656036 | 10.718514 |
| ER+ Breast cancer (Combined Oncoarray; iCOGS; GWAS meta analysis) \|\| id:ieu-a-1127 | Salad / raw vegetable intake \|\| id:ukb-b-1996 | Weighted median | 16 | 0.0852127 | 0.3127233 | 0.7852484 | 1.0889487 | 0.5899456 | 2.0100314 |
| ER+ Breast cancer (Combined Oncoarray; iCOGS; GWAS meta analysis) \|\| id:ieu-a-1127 | Salad / raw vegetable intake \|\| id:ukb-b-1996 | Inverse variance weighted | 16 | 0.0290544 | 0.246241 | 0.9060744 | 1.0294806 | 0.6353508 | 1.6681025 |
| ER+ Breast cancer (Combined Oncoarray; iCOGS; GWAS meta analysis) \|\| id:ieu-a-1127 | Salad / raw vegetable intake \|\| id:ukb-b-1996 | Simple mode | 16 | 0.3359324 | 0.6431261 | 0.6090601 | 1.3992444 | 0.3966921 | 4.9355275 |
| ER+ Breast cancer (Combined Oncoarray; iCOGS; GWAS meta analysis) \|\| id:ieu-a-1127 | Salad / raw vegetable intake \|\| id:ukb-b-1996 | Weighted mode | 16 | 0.0873489 | 0.6029766 | 0.8867477 | 1.0912774 | 0.3347119 | 3.5579443 |
| **Outcome** | **Exposure** | **Method** | **nsnp** | **b** | **se** | **pval** | **or** | **or_lci95** | **or_uci95** |
| ER+ Breast cancer (Combined Oncoarray; iCOGS; GWAS meta analysis) \|\| id:ieu-a-1127 | Non-oily fish intake \|\| id:ukb-b-17627 | MR Egger | 9 | -0.654946 | 1.524656 | 0.6804214 | 0.5194701 | 0.0261666 | 10.312736 |
| ER+ Breast cancer (Combined Oncoarray; iCOGS; GWAS meta analysis) \|\| id:ieu-a-1127 | Non-oily fish intake \|\| id:ukb-b-17627 | Weighted median | 9 | -0.12513 | 0.3090532 | 0.6855644 | 0.8823822 | 0.4814878 | 1.6170674 |
| ER+ Breast cancer (Combined Oncoarray; iCOGS; GWAS meta analysis) \|\| id:ieu-a-1127 | Non-oily fish intake \|\| id:ukb-b-17627 | Inverse variance weighted | 9 | -0.241871 | 0.2967201 | 0.4149877 | 0.7851577 | 0.4389182 | 1.4045271 |
| ER+ Breast cancer (Combined Oncoarray; iCOGS; GWAS meta analysis) \|\| id:ieu-a-1127 | Non-oily fish intake \|\| id:ukb-b-17627 | Simple mode | 9 | -0.092237 | 0.4509678 | 0.843046 | 0.911889 | 0.3767649 | 2.2070568 |
| ER+ Breast cancer (Combined Oncoarray; iCOGS; GWAS meta analysis) \|\| id:ieu-a-1127 | Non-oily fish intake \|\| id:ukb-b-17627 | Weighted mode | 9 | -0.130777 | 0.4201874 | 0.7635783 | 0.8774138 | 0.3850647 | 1.999287 |
| **Outcome** | **Exposure** | **Method** | **nsnp** | **b** | **se** | **pval** | **or** | **or_lci95** | **or_uci95** |
| ER+ Breast cancer (Combined Oncoarray; iCOGS; GWAS meta analysis) \|\| id:ieu-a-1127 | Oily fish intake \|\| id:ukb-b-2209 | MR Egger | 58 | -0.706142 | 0.6973964 | 0.3156346 | 0.4935445 | 0.1258029 | 1.9362531 |
| ER+ Breast cancer (Combined Oncoarray; iCOGS; GWAS meta analysis) \|\| id:ieu-a-1127 | Oily fish intake \|\| id:ukb-b-2209 | Weighted median | 58 | -0.177743 | 0.1268661 | 0.1612047 | 0.8371572 | 0.6528545 | 1.0734891 |
| ER+ Breast cancer (Combined Oncoarray; iCOGS; GWAS meta analysis) \|\| id:ieu-a-1127 | Oily fish intake \|\| id:ukb-b-2209 | Inverse variance weighted | 58 | -0.320042 | 0.1567514 | 0.0411797 | 0.7261187 | 0.5340454 | 0.9872726 |
| ER+ Breast cancer (Combined Oncoarray; iCOGS; GWAS meta analysis) \|\| id:ieu-a-1127 | Oily fish intake \|\| id:ukb-b-2209 | Simple mode | 58 | -0.194201 | 0.296298 | 0.5148309 | 0.8234923 | 0.460729 | 1.4718838 |
| ER+ Breast cancer (Combined Oncoarray; iCOGS; GWAS meta analysis) \|\| id:ieu-a-1127 | Oily fish intake \|\| id:ukb-b-2209 | Weighted mode | 58 | -0.297314 | 0.3019566 | 0.3289702 | 0.7428111 | 0.4110056 | 1.3424837 |
| **Outcome** | **Exposure** | **Method** | **nsnp** | **b** | **se** | **pval** | **or** | **or_lci95** | **or_uci95** |
| ER+ Breast cancer (Combined Oncoarray; iCOGS; GWAS meta analysis) \|\| id:ieu-a-1127 | Cereal intake \|\| id:ukb-b-15926 | MR Egger | 38 | -0.123695 | 0.5716395 | 0.8299081 | 0.8836493 | 0.2881978 | 2.7093761 |
| ER+ Breast cancer (Combined Oncoarray; iCOGS; GWAS meta analysis) \|\| id:ieu-a-1127 | Cereal intake \|\| id:ukb-b-15926 | Weighted median | 38 | 0.0623713 | 0.1602555 | 0.6971289 | 1.0643575 | 0.777455 | 1.4571349 |
| ER+ Breast cancer (Combined Oncoarray; iCOGS; GWAS meta analysis) \|\| id:ieu-a-1127 | Cereal intake \|\| id:ukb-b-15926 | Inverse variance weighted | 38 | 0.1302272 | 0.1329982 | 0.3274982 | 1.1390872 | 0.8777011 | 1.4783162 |
| ER+ Breast cancer (Combined Oncoarray; iCOGS; GWAS meta analysis) \|\| id:ieu-a-1127 | Cereal intake \|\| id:ukb-b-15926 | Simple mode | 38 | -0.020437 | 0.3079117 | 0.9474381 | 0.9797704 | 0.535827 | 1.7915296 |
| ER+ Breast cancer (Combined Oncoarray; iCOGS; GWAS meta analysis) \|\| id:ieu-a-1127 | Cereal intake \|\| id:ukb-b-15926 | Weighted mode | 38 | 0.0388515 | 0.2276867 | 0.8654398 | 1.0396161 | 0.6653683 | 1.6243659 |
| **Outcome** | **Exposure** | **Method** | **nsnp** | **b** | **se** | **pval** | **or** | **or_lci95** | **or_uci95** |
| ER+ Breast cancer (Combined Oncoarray; iCOGS; GWAS meta analysis) \|\| id:ieu-a-1127 | Salted nuts intake | MR Egger | 22 | 0.0991394 | 0.39818 | 0.8059142 | 1.1042202 | 0.5059622 | 2.4098685 |
| ER+ Breast cancer (Combined Oncoarray; iCOGS; GWAS meta analysis) \|\| id:ieu-a-1127 | Salted nuts intake | Weighted median | 22 | 0.3447868 | 0.2985656 | 0.2481679 | 1.4116889 | 0.7863119 | 2.5344465 |
| ER+ Breast cancer (Combined Oncoarray; iCOGS; GWAS meta analysis) \|\| id:ieu-a-1127 | Salted nuts intake | Inverse variance weighted | 22 | 0.1589663 | 0.2188231 | 0.4675567 | 1.1722984 | 0.7634352 | 1.8001312 |
| ER+ Breast cancer (Combined Oncoarray; iCOGS; GWAS meta analysis) \|\| id:ieu-a-1127 | Salted nuts intake | Simple mode | 22 | 0.5692249 | 0.5556024 | 0.3172482 | 1.7668969 | 0.5946657 | 5.2498823 |
| ER+ Breast cancer (Combined Oncoarray; iCOGS; GWAS meta analysis) \|\| id:ieu-a-1127 | Salted nuts intake | Weighted mode | 22 | 0.5692249 | 0.5329648 | 0.2976293 | 1.7668969 | 0.6216449 | 5.0220383 |
| **Outcome** | **Exposure** | **Method** | **nsnp** | **b** | **se** | **pval** | **or** | **or_lci95** | **or_uci95** |
| ER+ Breast cancer (Combined Oncoarray; iCOGS; GWAS meta analysis) \|\| id:ieu-a-1127 | Unsalted nuts intake | MR Egger | 15 | 0.8468843 | 0.446006 | 0.080008 | 2.3323684 | 0.9730814 | 5.5904291 |
| ER+ Breast cancer (Combined Oncoarray; iCOGS; GWAS meta analysis) \|\| id:ieu-a-1127 | Unsalted nuts intake | Weighted median | 15 | -0.137822 | 0.2618922 | 0.598712 | 0.871254 | 0.521456 | 1.4557002 |
| ER+ Breast cancer (Combined Oncoarray; iCOGS; GWAS meta analysis) \|\| id:ieu-a-1127 | Unsalted nuts intake | Inverse variance weighted | 15 | 0.146636 | 0.2375408 | 0.5370312 | 1.1579324 | 0.7269162 | 1.8445144 |
| ER+ Breast cancer (Combined Oncoarray; iCOGS; GWAS meta analysis) \|\| id:ieu-a-1127 | Unsalted nuts intake | Simple mode | 15 | -0.322603 | 0.583184 | 0.5888682 | 0.7242611 | 0.2309292 | 2.2714931 |
| ER+ Breast cancer (Combined Oncoarray; iCOGS; GWAS meta analysis) \|\| id:ieu-a-1127 | Unsalted nuts intake | Weighted mode | 15 | -0.274415 | 0.4500632 | 0.5518116 | 0.7600164 | 0.314573 | 1.836219 |
| **Outcome** | **Exposure** | **Method** | **nsnp** | **b** | **se** | **pval** | **or** | **or_lci95** | **or_uci95** |
| ER+ Breast cancer (Combined Oncoarray; iCOGS; GWAS meta analysis) \|\| id:ieu-a-1127 | Type of fat/oil used in cooking: Olive oil | MR Egger | 8 | -1.490669 | 0.7184651 | 0.0833326 | 0.225222 | 0.055086 | 0.9208326 |
| ER+ Breast cancer (Combined Oncoarray; iCOGS; GWAS meta analysis) \|\| id:ieu-a-1127 | Type of fat/oil used in cooking: Olive oil | Weighted median | 8 | -0.345599 | 0.3096907 | 0.2644438 | 0.7077961 | 0.3857395 | 1.2987401 |
| ER+ Breast cancer (Combined Oncoarray; iCOGS; GWAS meta analysis) \|\| id:ieu-a-1127 | Type of fat/oil used in cooking: Olive oil | Inverse variance weighted | 8 | -0.317288 | 0.2453219 | 0.1958889 | 0.7281211 | 0.450175 | 1.177676 |
| ER+ Breast cancer (Combined Oncoarray; iCOGS; GWAS meta analysis) \|\| id:ieu-a-1127 | Type of fat/oil used in cooking: Olive oil | Simple mode | 8 | -0.561423 | 0.5404743 | 0.3334603 | 0.5703968 | 0.1977498 | 1.6452736 |
| ER+ Breast cancer (Combined Oncoarray; iCOGS; GWAS meta analysis) \|\| id:ieu-a-1127 | Type of fat/oil used in cooking: Olive oil | Weighted mode | 8 | -0.5268 | 0.5074888 | 0.3337675 | 0.5904916 | 0.2183889 | 1.596603 |
| Notes: or = odds ratio; or_lci95 =the lower limit of 95% confidence interval; or_uci95= the upper limit of 95% confidence interval | | | | | | | | | |

**Supplemental Table 5.** **Causal effects of 7 dietary habits on ER- BC risks using MR-Egger, weighted median, inverse variance weighted, simple mode, and weighted mode methods**

| **Outcome** | **Exposure** | **Method** | **nsnp** | **b** | **se** | **pval** | **or** | **or_lci95** | **or_uci95** |
| --- | --- | --- | --- | --- | --- | --- | --- | --- | --- |
| ER- Breast cancer (Combined Oncoarray; iCOGS; GWAS meta analysis) \|\| id:ieu-a-1128 | Fresh fruit intake \|\| id:ukb-b-3881 | MR Egger | 47 | -0.777854 | 0.7401961 | 0.2989264 | 0.4593906 | 0.1076749 | 1.9599716 |
| ER- Breast cancer (Combined Oncoarray; iCOGS; GWAS meta analysis) \|\| id:ieu-a-1128 | Fresh fruit intake \|\| id:ukb-b-3881 | Weighted median | 47 | -0.709574 | 0.3122535 | 0.0230606 | 0.4918536 | 0.2667106 | 0.9070503 |
| ER- Breast cancer (Combined Oncoarray; iCOGS; GWAS meta analysis) \|\| id:ieu-a-1128 | Fresh fruit intake \|\| id:ukb-b-3881 | Inverse variance weighted | 47 | -0.428295 | 0.2187613 | 0.0502514 | 0.6516193 | 0.424405 | 1.0004775 |
| ER- Breast cancer (Combined Oncoarray; iCOGS; GWAS meta analysis) \|\| id:ieu-a-1128 | Fresh fruit intake \|\| id:ukb-b-3881 | Simple mode | 47 | -0.672233 | 0.7107663 | 0.3491995 | 0.5105673 | 0.1267758 | 2.0562194 |
| ER- Breast cancer (Combined Oncoarray; iCOGS; GWAS meta analysis) \|\| id:ieu-a-1128 | Fresh fruit intake \|\| id:ukb-b-3881 | Weighted mode | 47 | -0.761378 | 0.5159727 | 0.1468594 | 0.4670223 | 0.1698763 | 1.2839335 |
| **Outcome** | **Exposure** | **Method** | **nsnp** | **b** | **se** | **pval** | **or** | **or_lci95** | **or_uci95** |
| ER- Breast cancer (Combined Oncoarray; iCOGS; GWAS meta analysis) \|\| id:ieu-a-1128 | Dried fruit intake \|\| id:ukb-b-16576 | MR Egger | 39 | -0.542051 | 0.8185703 | 0.5119519 | 0.5815544 | 0.1168986 | 2.8931534 |
| ER- Breast cancer (Combined Oncoarray; iCOGS; GWAS meta analysis) \|\| id:ieu-a-1128 | Dried fruit intake \|\| id:ukb-b-16576 | Weighted median | 39 | -0.690462 | 0.2523634 | 0.0062194 | 0.5013445 | 0.3057174 | 0.8221525 |
| ER- Breast cancer (Combined Oncoarray; iCOGS; GWAS meta analysis) \|\| id:ieu-a-1128 | Dried fruit intake \|\| id:ukb-b-16576 | Inverse variance weighted | 39 | -0.733729 | 0.1763762 | 3.18E-05 | 0.4801154 | 0.3397905 | 0.6783909 |
| ER- Breast cancer (Combined Oncoarray; iCOGS; GWAS meta analysis) \|\| id:ieu-a-1128 | Dried fruit intake \|\| id:ukb-b-16576 | Simple mode | 39 | -0.753216 | 0.455649 | 0.1065581 | 0.4708501 | 0.1927642 | 1.1501087 |
| ER- Breast cancer (Combined Oncoarray; iCOGS; GWAS meta analysis) \|\| id:ieu-a-1128 | Dried fruit intake \|\| id:ukb-b-16576 | Weighted mode | 39 | -0.716415 | 0.4322691 | 0.1056865 | 0.4885006 | 0.209368 | 1.1397771 |
| **Outcome** | **Exposure** | **Method** | **nsnp** | **b** | **se** | **pval** | **or** | **or_lci95** | **or_uci95** |
| ER- Breast cancer (Combined Oncoarray; iCOGS; GWAS meta analysis) \|\| id:ieu-a-1128 | Cooked vegetable intake \|\| id:ukb-b-8089 | MR Egger | 16 | 2.9394363 | 3.7213725 | 0.4427718 | 18.905187 | 0.0128495 | 27814.879 |
| ER- Breast cancer (Combined Oncoarray; iCOGS; GWAS meta analysis) \|\| id:ieu-a-1128 | Cooked vegetable intake \|\| id:ukb-b-8089 | Weighted median | 16 | -0.170942 | 0.4261276 | 0.6883082 | 0.8428705 | 0.3656232 | 1.9430678 |
| ER- Breast cancer (Combined Oncoarray; iCOGS; GWAS meta analysis) \|\| id:ieu-a-1128 | Cooked vegetable intake \|\| id:ukb-b-8089 | Inverse variance weighted | 16 | -0.169203 | 0.3464546 | 0.6252766 | 0.8443371 | 0.4281617 | 1.6650372 |
| ER- Breast cancer (Combined Oncoarray; iCOGS; GWAS meta analysis) \|\| id:ieu-a-1128 | Cooked vegetable intake \|\| id:ukb-b-8089 | Simple mode | 16 | -0.332929 | 0.6462599 | 0.6139471 | 0.7168209 | 0.2019775 | 2.5440065 |
| ER- Breast cancer (Combined Oncoarray; iCOGS; GWAS meta analysis) \|\| id:ieu-a-1128 | Cooked vegetable intake \|\| id:ukb-b-8089 | Weighted mode | 16 | -0.31258 | 0.6600576 | 0.6426288 | 0.731557 | 0.20063 | 2.6674758 |
| **Outcome** | **Exposure** | **Method** | **nsnp** | **b** | **se** | **pval** | **or** | **or_lci95** | **or_uci95** |
| ER- Breast cancer (Combined Oncoarray; iCOGS; GWAS meta analysis) \|\| id:ieu-a-1128 | Salad / raw vegetable intake \|\| id:ukb-b-1996 | MR Egger | 18 | -1.741785 | 1.6927713 | 0.3188057 | 0.1752074 | 0.006348 | 4.8358006 |
| ER- Breast cancer (Combined Oncoarray; iCOGS; GWAS meta analysis) \|\| id:ieu-a-1128 | Salad / raw vegetable intake \|\| id:ukb-b-1996 | Weighted median | 18 | 0.2564819 | 0.4421176 | 0.5618328 | 1.2923754 | 0.5433136 | 3.0741623 |
| ER- Breast cancer (Combined Oncoarray; iCOGS; GWAS meta analysis) \|\| id:ieu-a-1128 | Salad / raw vegetable intake \|\| id:ukb-b-1996 | Inverse variance weighted | 18 | 0.3679532 | 0.3356465 | 0.2729684 | 1.4447744 | 0.748328 | 2.7893826 |
| ER- Breast cancer (Combined Oncoarray; iCOGS; GWAS meta analysis) \|\| id:ieu-a-1128 | Salad / raw vegetable intake \|\| id:ukb-b-1996 | Simple mode | 18 | -0.139797 | 0.7648873 | 0.8571414 | 0.8695343 | 0.1941787 | 3.8937844 |
| ER- Breast cancer (Combined Oncoarray; iCOGS; GWAS meta analysis) \|\| id:ieu-a-1128 | Salad / raw vegetable intake \|\| id:ukb-b-1996 | Weighted mode | 18 | 0.0181488 | 0.6932391 | 0.9794189 | 1.0183144 | 0.2616886 | 3.9625884 |
| **Outcome** | **Exposure** | **Method** | **nsnp** | **b** | **se** | **pval** | **or** | **or_lci95** | **or_uci95** |
| ER- Breast cancer (Combined Oncoarray; iCOGS; GWAS meta analysis) \|\| id:ieu-a-1128 | Non-oily fish intake \|\| id:ukb-b-17627 | MR Egger | 9 | -0.357283 | 1.7643915 | 0.8452883 | 0.6995744 | 0.0220269 | 22.218476 |
| ER- Breast cancer (Combined Oncoarray; iCOGS; GWAS meta analysis) \|\| id:ieu-a-1128 | Non-oily fish intake \|\| id:ukb-b-17627 | Weighted median | 9 | -0.097162 | 0.4712176 | 0.8366405 | 0.9074094 | 0.3603253 | 2.2851345 |
| ER- Breast cancer (Combined Oncoarray; iCOGS; GWAS meta analysis) \|\| id:ieu-a-1128 | Non-oily fish intake \|\| id:ukb-b-17627 | Inverse variance weighted | 9 | -0.069352 | 0.3643596 | 0.8490435 | 0.9329983 | 0.4568059 | 1.9055924 |
| ER- Breast cancer (Combined Oncoarray; iCOGS; GWAS meta analysis) \|\| id:ieu-a-1128 | Non-oily fish intake \|\| id:ukb-b-17627 | Simple mode | 9 | -0.395724 | 0.7244009 | 0.5997746 | 0.6731926 | 0.1627484 | 2.7845936 |
| ER- Breast cancer (Combined Oncoarray; iCOGS; GWAS meta analysis) \|\| id:ieu-a-1128 | Non-oily fish intake \|\| id:ukb-b-17627 | Weighted mode | 9 | 0.0562467 | 0.6521761 | 0.9333915 | 1.0578586 | 0.2946348 | 3.7981417 |
| **Outcome** | **Exposure** | **Method** | **nsnp** | **b** | **se** | **pval** | **or** | **or_lci95** | **or_uci95** |
| ER- Breast cancer (Combined Oncoarray; iCOGS; GWAS meta analysis) \|\| id:ieu-a-1128 | Oily fish intake \|\| id:ukb-b-2209 | MR Egger | 55 | 0.9082272 | 0.6983669 | 0.1990558 | 2.4799222 | 0.6309227 | 9.7476506 |
| ER- Breast cancer (Combined Oncoarray; iCOGS; GWAS meta analysis) \|\| id:ieu-a-1128 | Oily fish intake \|\| id:ukb-b-2209 | Weighted median | 55 | 0.0351697 | 0.1946263 | 0.8566001 | 1.0357955 | 0.7073018 | 1.516852 |
| ER- Breast cancer (Combined Oncoarray; iCOGS; GWAS meta analysis) \|\| id:ieu-a-1128 | Oily fish intake \|\| id:ukb-b-2209 | Inverse variance weighted | 55 | 0.0399077 | 0.1562075 | 0.7983532 | 1.0407147 | 0.7662407 | 1.4135077 |
| ER- Breast cancer (Combined Oncoarray; iCOGS; GWAS meta analysis) \|\| id:ieu-a-1128 | Oily fish intake \|\| id:ukb-b-2209 | Simple mode | 55 | -0.382331 | 0.5434615 | 0.4847598 | 0.6822691 | 0.2351538 | 1.9795181 |
| ER- Breast cancer (Combined Oncoarray; iCOGS; GWAS meta analysis) \|\| id:ieu-a-1128 | Oily fish intake \|\| id:ukb-b-2209 | Weighted mode | 55 | 0.5742633 | 0.4820975 | 0.2387937 | 1.7758219 | 0.6902871 | 4.5684519 |
| **Outcome** | **Exposure** | **Method** | **nsnp** | **b** | **se** | **pval** | **or** | **or_lci95** | **or_uci95** |
| ER- Breast cancer (Combined Oncoarray; iCOGS; GWAS meta analysis) \|\| id:ieu-a-1128 | Cereal intake \|\| id:ukb-b-15926 | MR Egger | 39 | -0.594984 | 0.7076357 | 0.4058577 | 0.5515712 | 0.1378002 | 2.2077668 |
| ER- Breast cancer (Combined Oncoarray; iCOGS; GWAS meta analysis) \|\| id:ieu-a-1128 | Cereal intake \|\| id:ukb-b-15926 | Weighted median | 39 | -0.188463 | 0.2311234 | 0.4148321 | 0.8282315 | 0.5265208 | 1.3028306 |
| ER- Breast cancer (Combined Oncoarray; iCOGS; GWAS meta analysis) \|\| id:ieu-a-1128 | Cereal intake \|\| id:ukb-b-15926 | Inverse variance weighted | 39 | -0.11875 | 0.1637781 | 0.4684107 | 0.8880296 | 0.6441941 | 1.22416 |
| ER- Breast cancer (Combined Oncoarray; iCOGS; GWAS meta analysis) \|\| id:ieu-a-1128 | Cereal intake \|\| id:ukb-b-15926 | Simple mode | 39 | -0.30452 | 0.44438 | 0.4973334 | 0.7374773 | 0.3086631 | 1.7620272 |
| ER- Breast cancer (Combined Oncoarray; iCOGS; GWAS meta analysis) \|\| id:ieu-a-1128 | Cereal intake \|\| id:ukb-b-15926 | Weighted mode | 39 | -0.320623 | 0.389966 | 0.4161029 | 0.7256969 | 0.3379166 | 1.5584793 |
| **Outcome** | **Exposure** | **Method** | **nsnp** | **b** | **se** | **pval** | **or** | **or_lci95** | **or_uci95** |
| ER- Breast cancer (Combined Oncoarray; iCOGS; GWAS meta analysis) \|\| id:ieu-a-1128 | Salted nuts intake | MR Egger | 22 | -0.992886 | 0.6397425 | 0.1363412 | 0.3705059 | 0.105739 | 1.2982396 |
| ER- Breast cancer (Combined Oncoarray; iCOGS; GWAS meta analysis) \|\| id:ieu-a-1128 | Salted nuts intake | Weighted median | 22 | -0.204294 | 0.4826882 | 0.6721182 | 0.8152225 | 0.3165218 | 2.0996585 |
| ER- Breast cancer (Combined Oncoarray; iCOGS; GWAS meta analysis) \|\| id:ieu-a-1128 | Salted nuts intake | Inverse variance weighted | 22 | -0.003876 | 0.3781192 | 0.9918217 | 0.9961317 | 0.4747393 | 2.0901544 |
| ER- Breast cancer (Combined Oncoarray; iCOGS; GWAS meta analysis) \|\| id:ieu-a-1128 | Salted nuts intake | Simple mode | 22 | -0.165388 | 0.9469789 | 0.8630292 | 0.8475649 | 0.1324611 | 5.4232254 |
| ER- Breast cancer (Combined Oncoarray; iCOGS; GWAS meta analysis) \|\| id:ieu-a-1128 | Salted nuts intake | Weighted mode | 22 | -0.307612 | 0.8398557 | 0.7178297 | 0.7352007 | 0.1417445 | 3.8133397 |
| **Outcome** | **Exposure** | **Method** | **nsnp** | **b** | **se** | **pval** | **or** | **or_lci95** | **or_uci95** |
| ER- Breast cancer (Combined Oncoarray; iCOGS; GWAS meta analysis) \|\| id:ieu-a-1128 | Unsalted nuts intake | MR Egger | 15 | 0.314788 | 0.6007931 | 0.6091278 | 1.3699688 | 0.421993 | 4.4475011 |
| ER- Breast cancer (Combined Oncoarray; iCOGS; GWAS meta analysis) \|\| id:ieu-a-1128 | Unsalted nuts intake | Weighted median | 15 | 0.1317295 | 0.3914356 | 0.7364718 | 1.1407997 | 0.5296789 | 2.4570055 |
| ER- Breast cancer (Combined Oncoarray; iCOGS; GWAS meta analysis) \|\| id:ieu-a-1128 | Unsalted nuts intake | Inverse variance weighted | 15 | 0.1654878 | 0.2856216 | 0.5623225 | 1.1799686 | 0.6741312 | 2.0653633 |
| ER- Breast cancer (Combined Oncoarray; iCOGS; GWAS meta analysis) \|\| id:ieu-a-1128 | Unsalted nuts intake | Simple mode | 15 | 0.2818267 | 0.5922122 | 0.6414948 | 1.3255489 | 0.4152356 | 4.2315254 |
| ER- Breast cancer (Combined Oncoarray; iCOGS; GWAS meta analysis) \|\| id:ieu-a-1128 | Unsalted nuts intake | Weighted mode | 15 | 0.2133459 | 0.5771709 | 0.7171814 | 1.2378128 | 0.3993532 | 3.8366554 |
| **Outcome** | **Exposure** | **Method** | **nsnp** | **b** | **se** | **pval** | **or** | **or_lci95** | **or_uci95** |
| ER- Breast cancer (Combined Oncoarray; iCOGS; GWAS meta analysis) \|\| id:ieu-a-1128 | Type of fat/oil used in cooking: Olive oil | MR Egger | 8 | -0.058737 | 1.0909129 | 0.958809 | 0.9429548 | 0.1111443 | 8.0000869 |
| ER- Breast cancer (Combined Oncoarray; iCOGS; GWAS meta analysis) \|\| id:ieu-a-1128 | Type of fat/oil used in cooking: Olive oil | Weighted median | 8 | -0.292677 | 0.4118118 | 0.4772672 | 0.7462634 | 0.3329284 | 1.6727593 |
| ER- Breast cancer (Combined Oncoarray; iCOGS; GWAS meta analysis) \|\| id:ieu-a-1128 | Type of fat/oil used in cooking: Olive oil | Inverse variance weighted | 8 | -0.138413 | 0.3345382 | 0.6790622 | 0.8707391 | 0.4519844 | 1.677462 |
| ER- Breast cancer (Combined Oncoarray; iCOGS; GWAS meta analysis) \|\| id:ieu-a-1128 | Type of fat/oil used in cooking: Olive oil | Simple mode | 8 | -0.377223 | 0.616476 | 0.5599436 | 0.6857629 | 0.204842 | 2.2957732 |
| ER- Breast cancer (Combined Oncoarray; iCOGS; GWAS meta analysis) \|\| id:ieu-a-1128 | Type of fat/oil used in cooking: Olive oil | Weighted mode | 8 | -0.356944 | 0.5878744 | 0.5629026 | 0.699812 | 0.2210918 | 2.2150836 |
| Notes: or = odds ratio; or_lci95 =the lower limit of 95% confidence interval; or_uci95= the upper limit of 95% confidence interval | | | | | | | | | |
